# Supplementary material for: Somatic selection shapes mutation accumulation in Citrus sinensis
Source: Plant Cell. 2026 Sep 18;38(10):koag288. doi: 10.1093/plcell/koag288 (PMC13690466; doi:10.1093/plcell/koag288)
Supplement: Supplementary file 1 [file koag288_supplementary_data.pdf]

Supplemental Figures

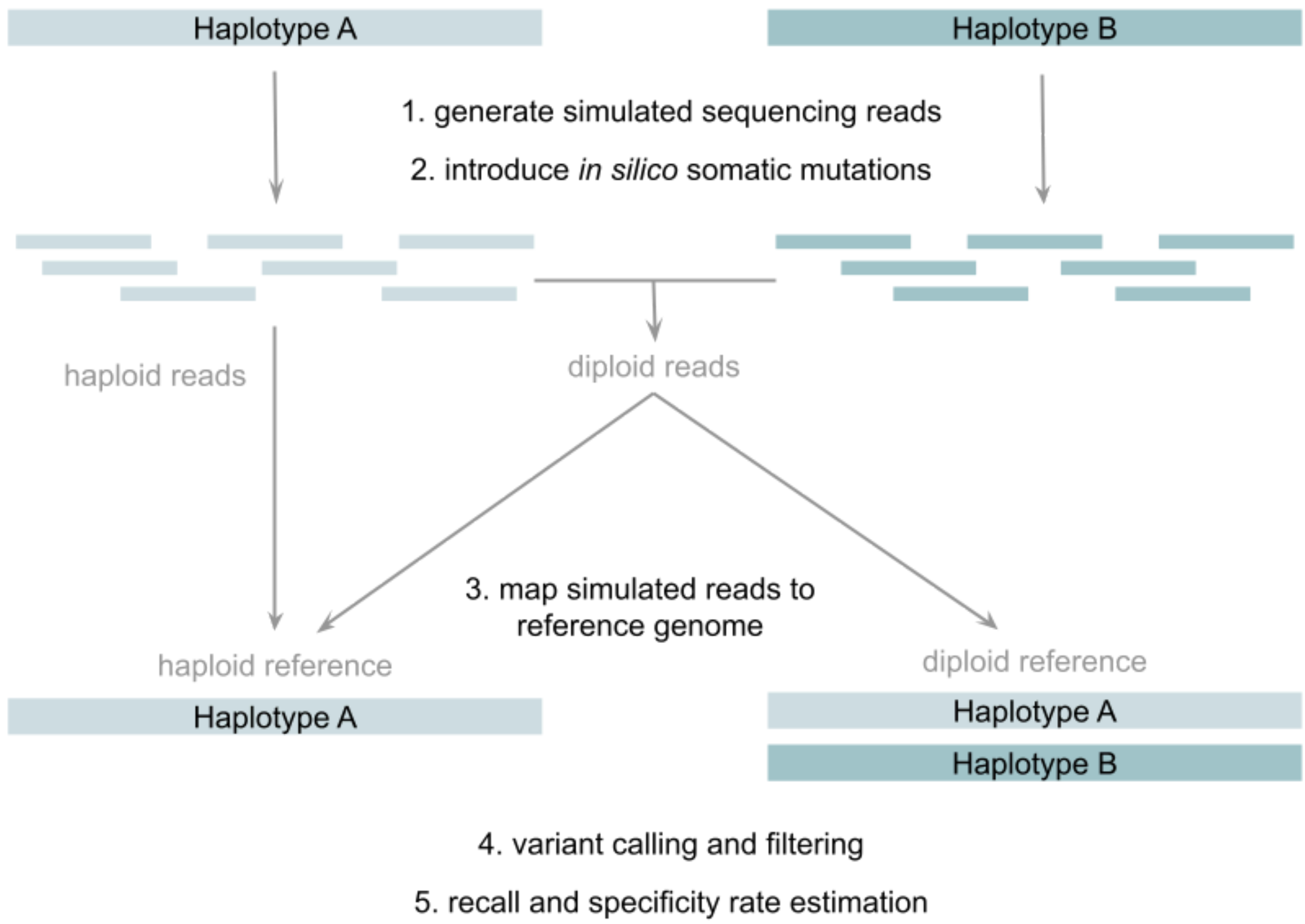

Figure S1. *In silico* somatic mutation analysis workflow.

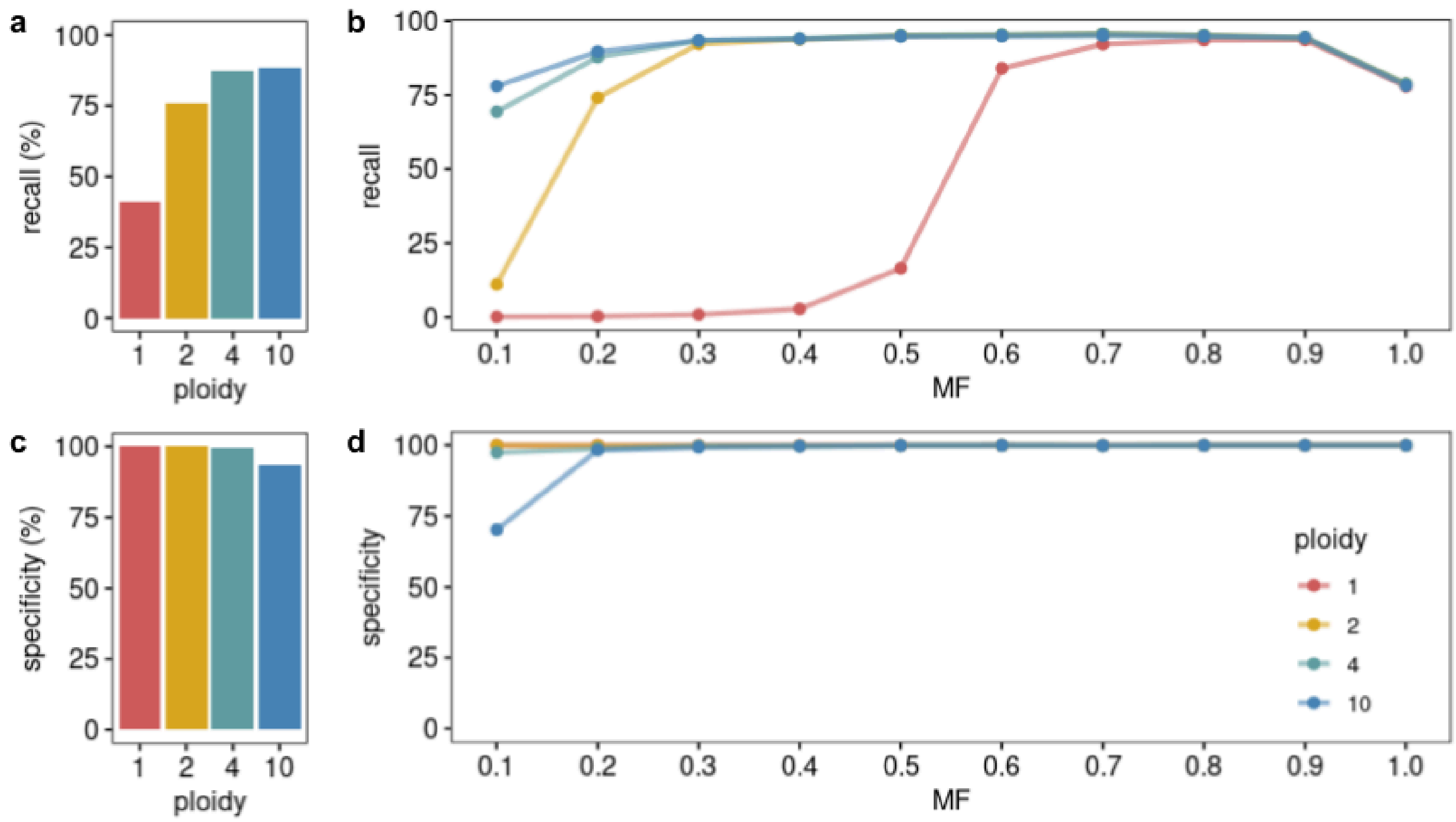

Figure S2. *In silico* somatic mutation detection rates. **(a-b)** The effect of increasing the GATK HaplotypeCaller ploidy parameter on the rate of recall for **(a)** overall *in silico* mutation detection and for **(b)** *in silico* mutation detection for MFs ranging from 0.1 to 1. The recall rate is the percentage of introduced *in silico* mutations which were called as variants. **(c-d)** The effect of increasing the GATK HaplotypeCaller ploidy parameter on the rate of specificity for **(c)** overall *in silico* mutation detection for overall and for **(d)** *in silico* mutation detection for MFs ranging from 0.1 to 1. The specificity rate is the percentage of variants called which were introduced *in silico* mutations.

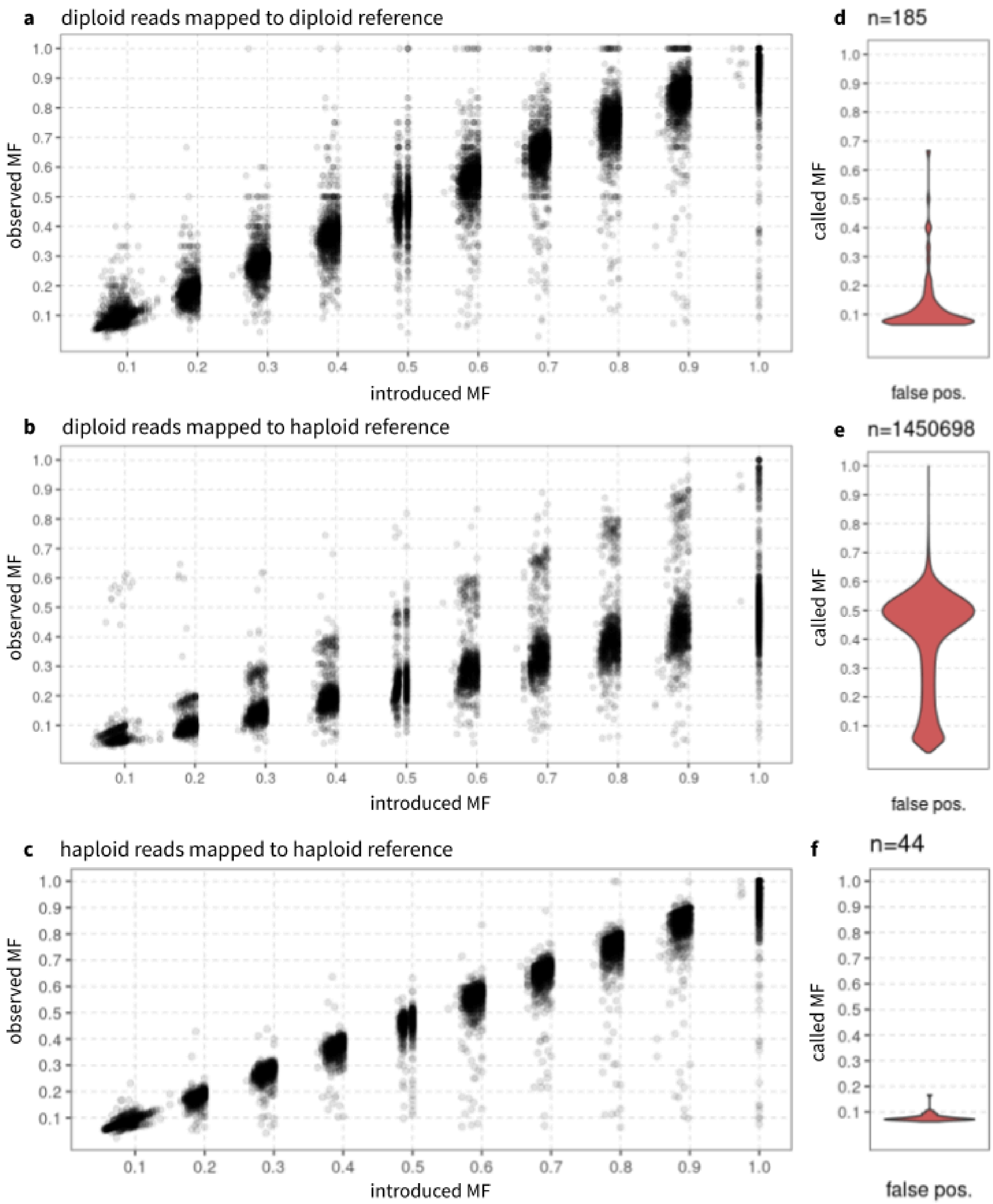

a diploid reads mapped to diploid reference
observed MF
introduced MF
d n=185
called MF
false pos.
b diploid reads mapped to haploid reference
observed MF
introduced MF
e n=1450698
called MF
false pos.
c haploid reads mapped to haploid reference
observed MF
introduced MF
f n=44
called MF
false pos.

Figure S3. The MFs of *in silico* somatic mutations. **(a-c)** The relationship between the introduced MF and the observed MF of *in silico* somatic mutations. Variants were identified using GATK HaplotypeCaller (with a ploidy parameter of 4) from **(a)** simulated reads from a diploid genome which were mapped to a diploid reference genome, **(b)** simulated reads from a diploid genome which were mapped to a haploid reference genome, and **(c)** simulated reads from a haploid genome which were mapped to a haploid reference genome. **(d-f)** The distribution of MFs of false positives identified using GATK HaplotypeCaller with a ploidy parameter of 4 from **(d)** simulated reads from a diploid genome which were mapped to a diploid reference genome, **(e)** simulated reads from a diploid genome which were mapped to a haploid reference genome, and **(f)** simulated reads from a haploid genome which were mapped to a haploid reference genome. The diploid reference genome refers to the 'Washington navel' genome assembly and the haploid reference genome refers to haplotype 1 of the same assembly.

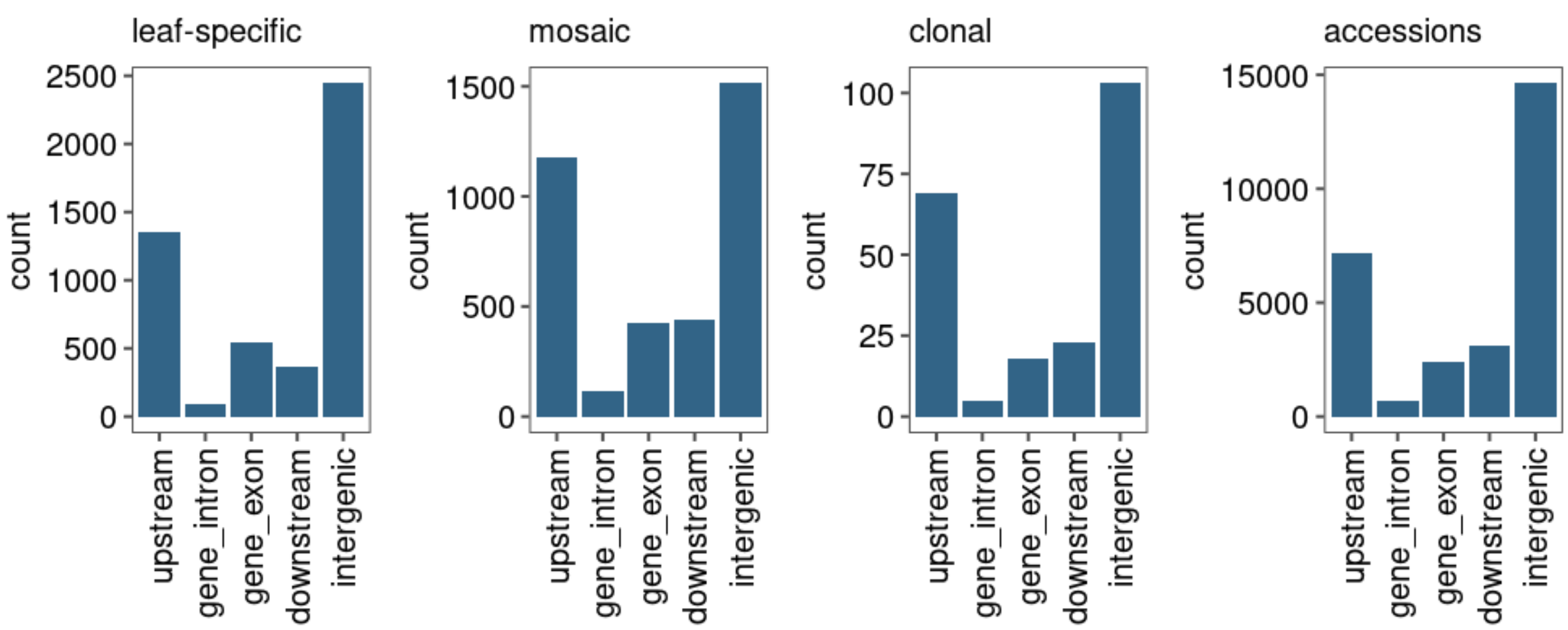

Figures S4. Somatic mutations by genomic feature. The total number of somatic mutations in genic and intergenic regions. Upstream and downstream regions include 5 Kb from the transcription start (or termination) site.

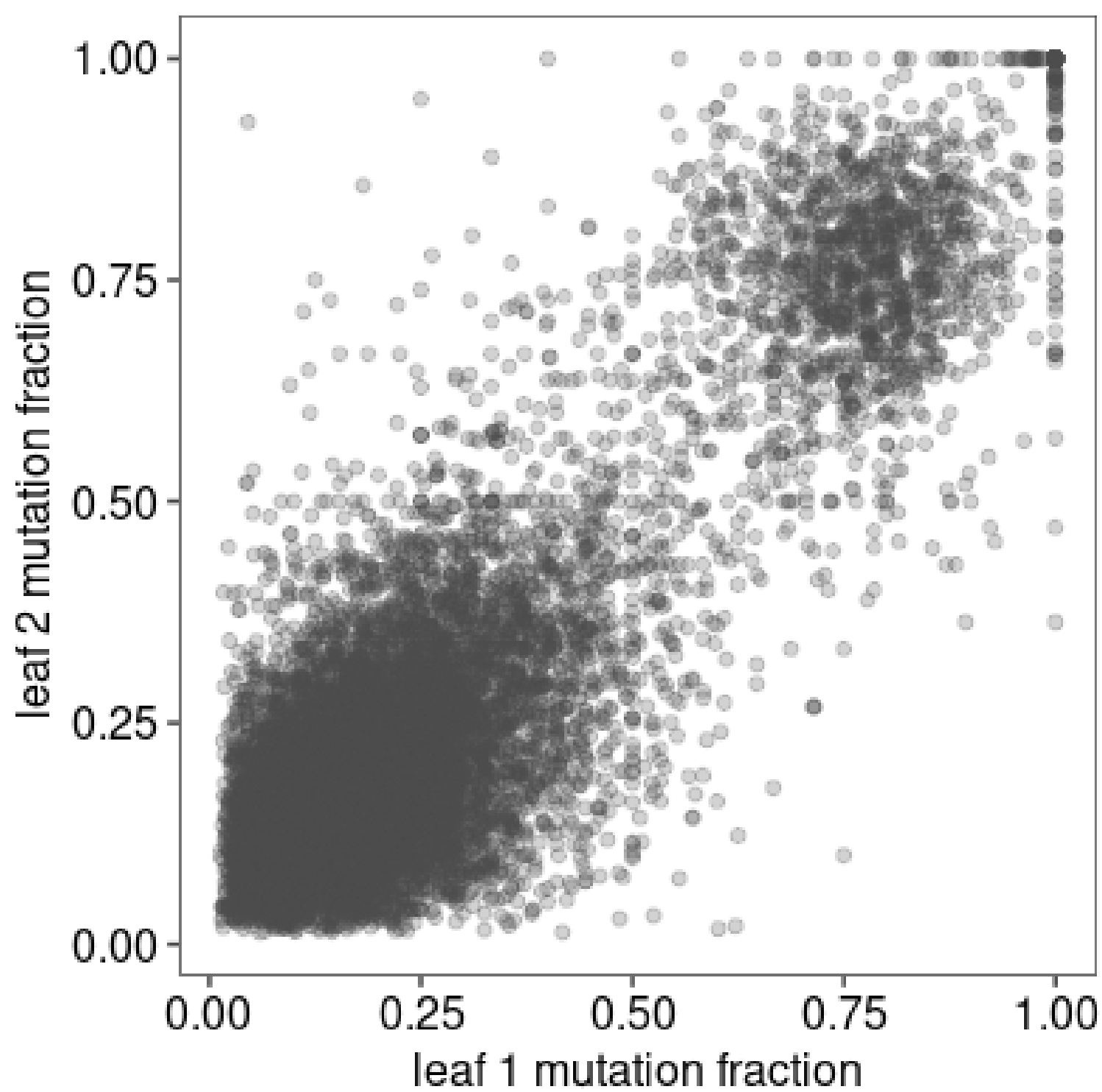

Figure S5. Concordance between the observed mutation fraction at adjacent leaves. Each point represents a mutation identified in adjacent leaves of a leaf-pair collected at one sampling location.

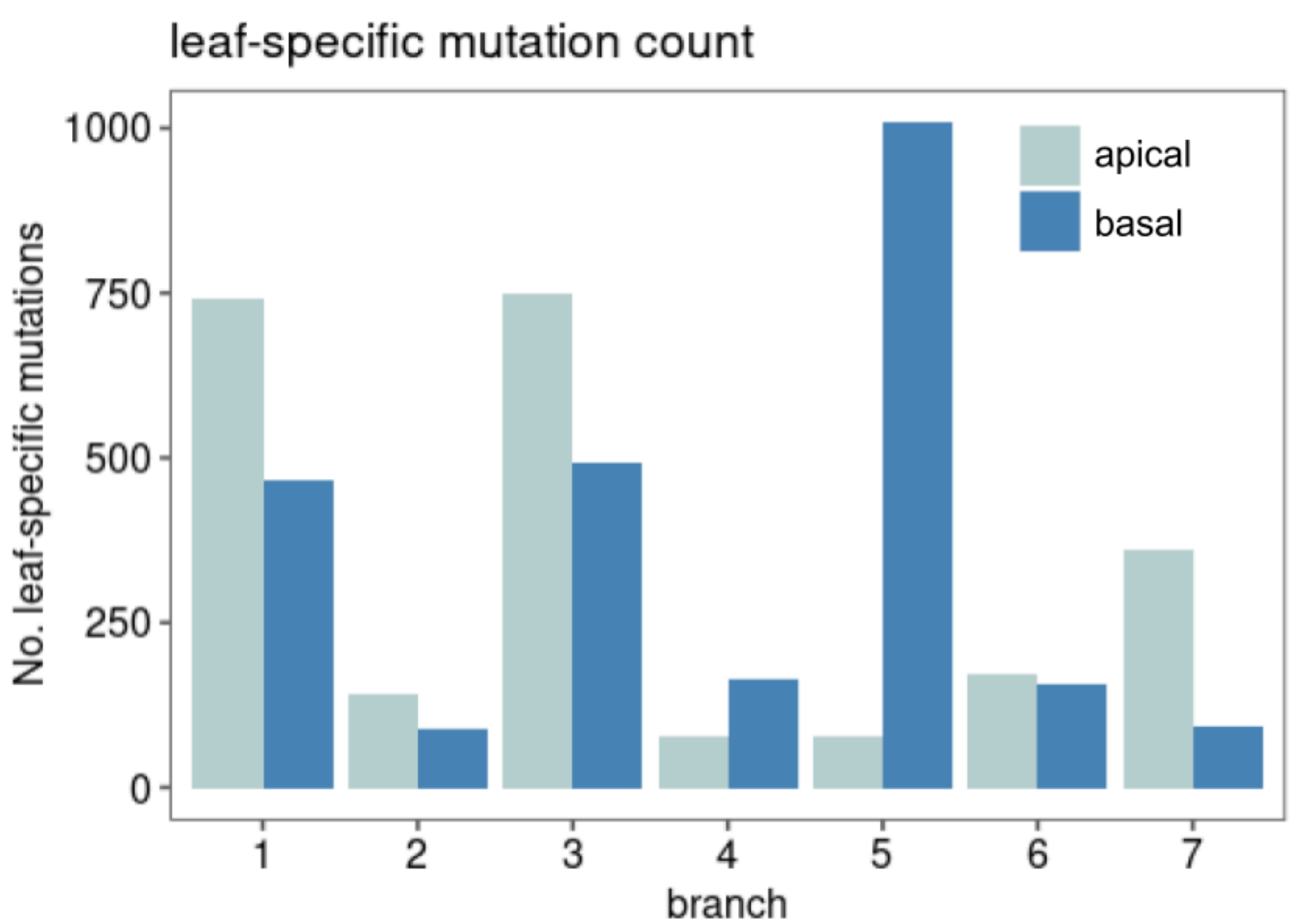

Figure S6. Number of leaf-specific somatic mutations detected at each sampling location.

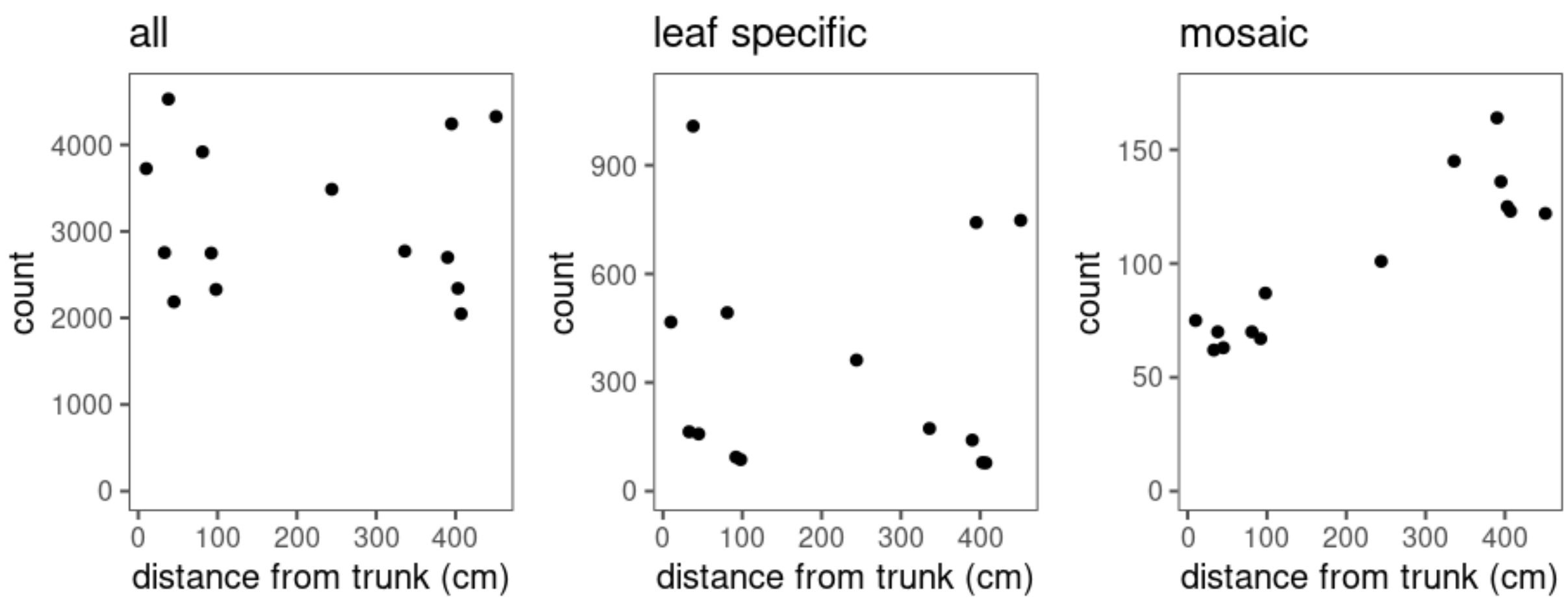

Figure S7. The relationship between physical distance (cm) and somatic mutation number per sample.

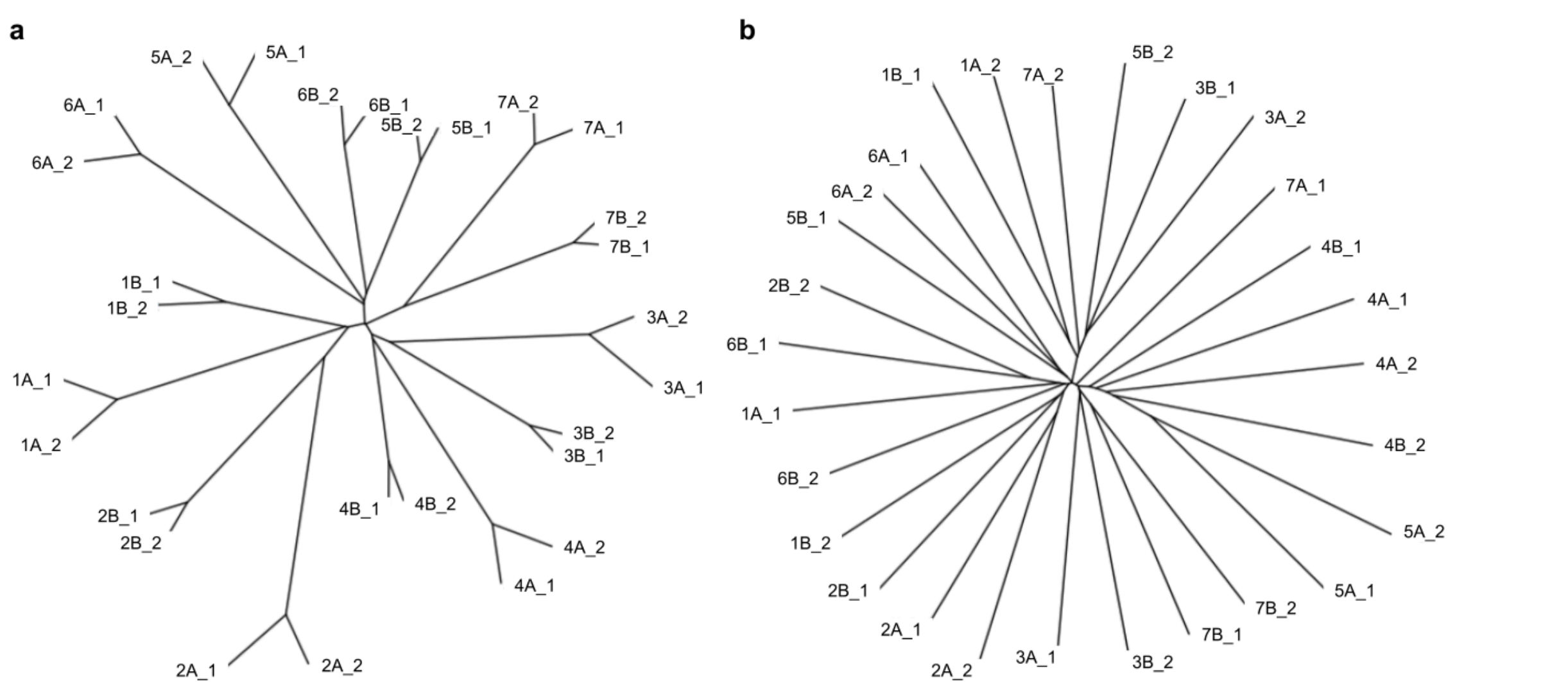

Figure S8. The phylogenetic trees constructed using the mutation fraction (MF) matrix of **(a)** completely-detected or **(b)** incompletely-detected mosaic somatic mutations.

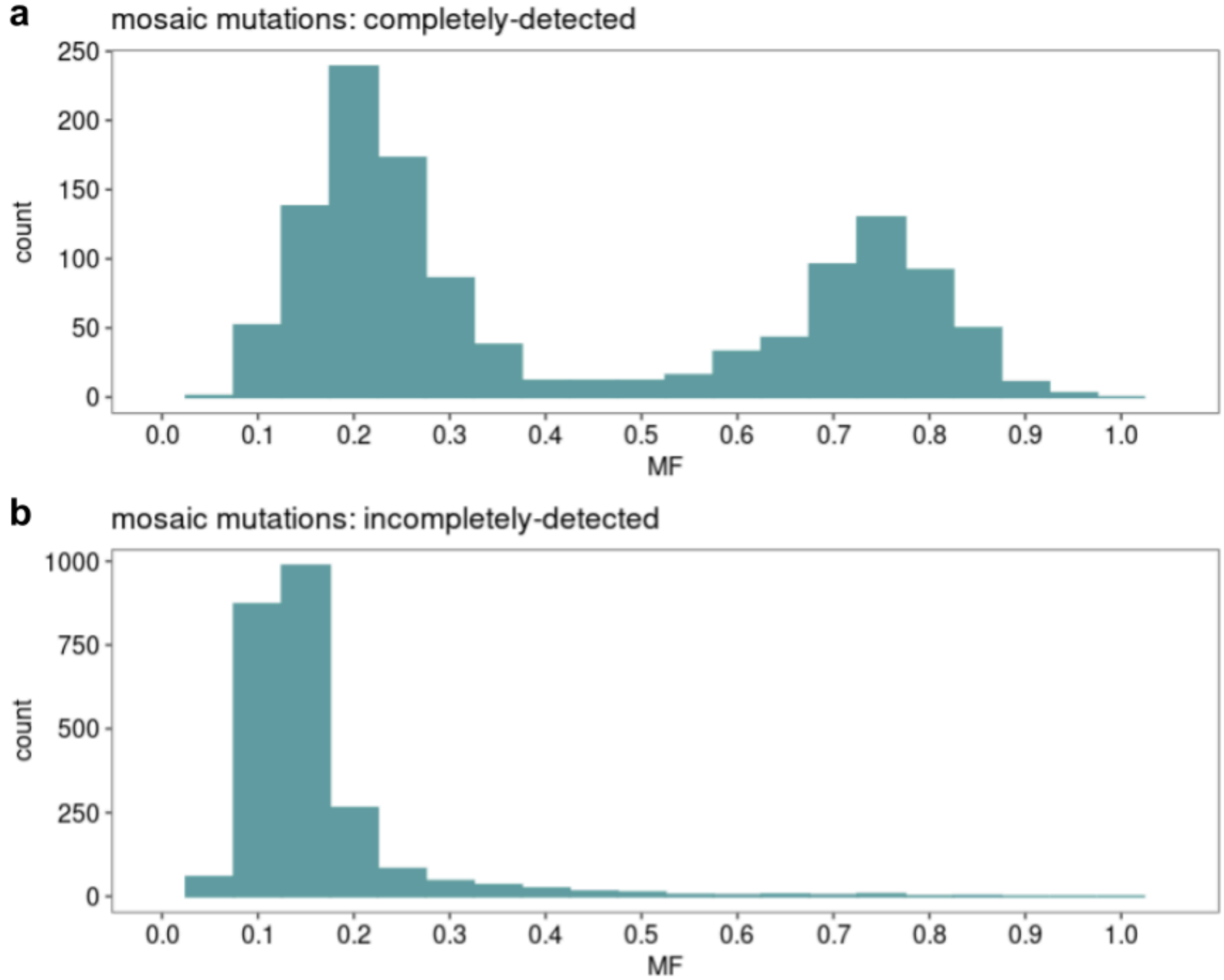

Figure S9. Mutation fraction (MF) distribution of **(a)** completely-detected and **(b)** incompletely-detected mosaic mutations.

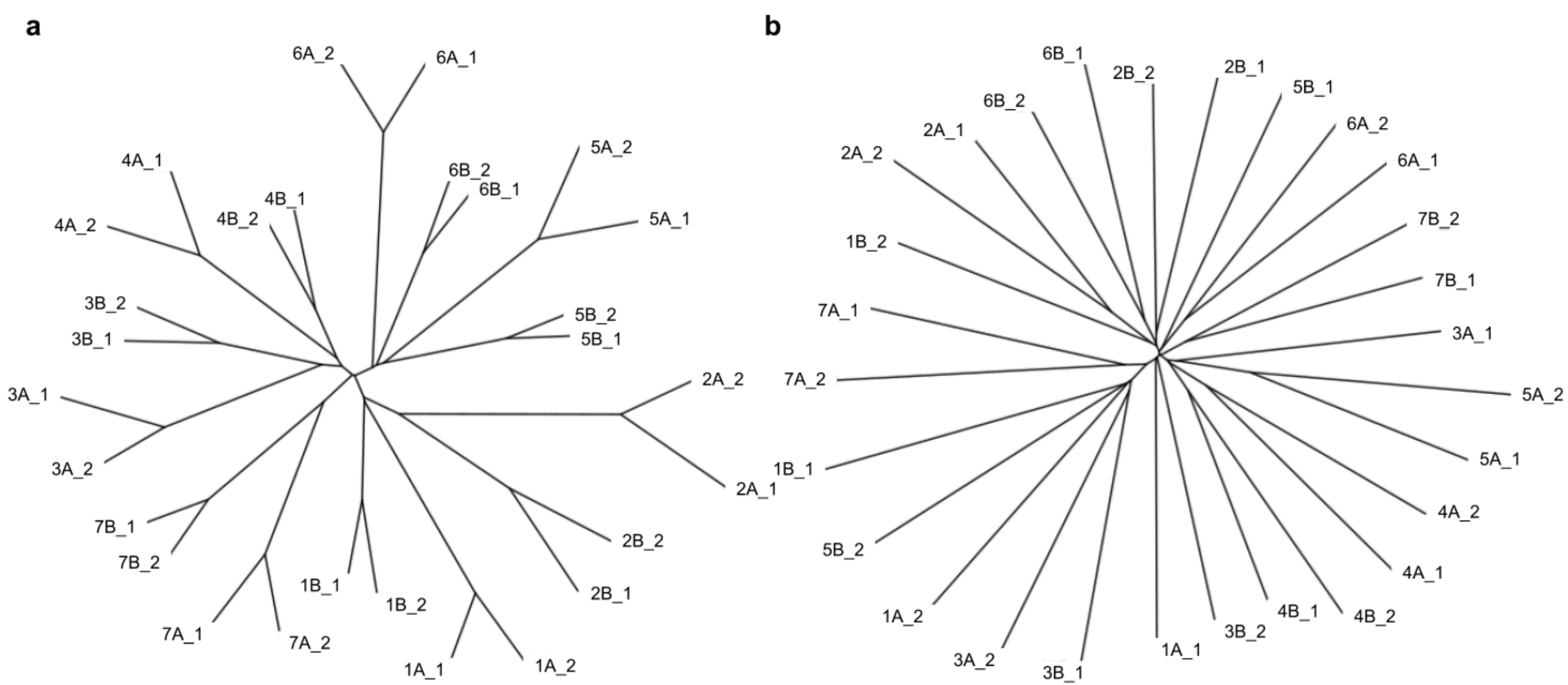

Figure S10. The phylogenetic trees constructed using the MF matrix of **(a)** high-fraction (MF > 0.5) or **(b)** low-fraction (MF < 0.5) mosaic somatic mutations.

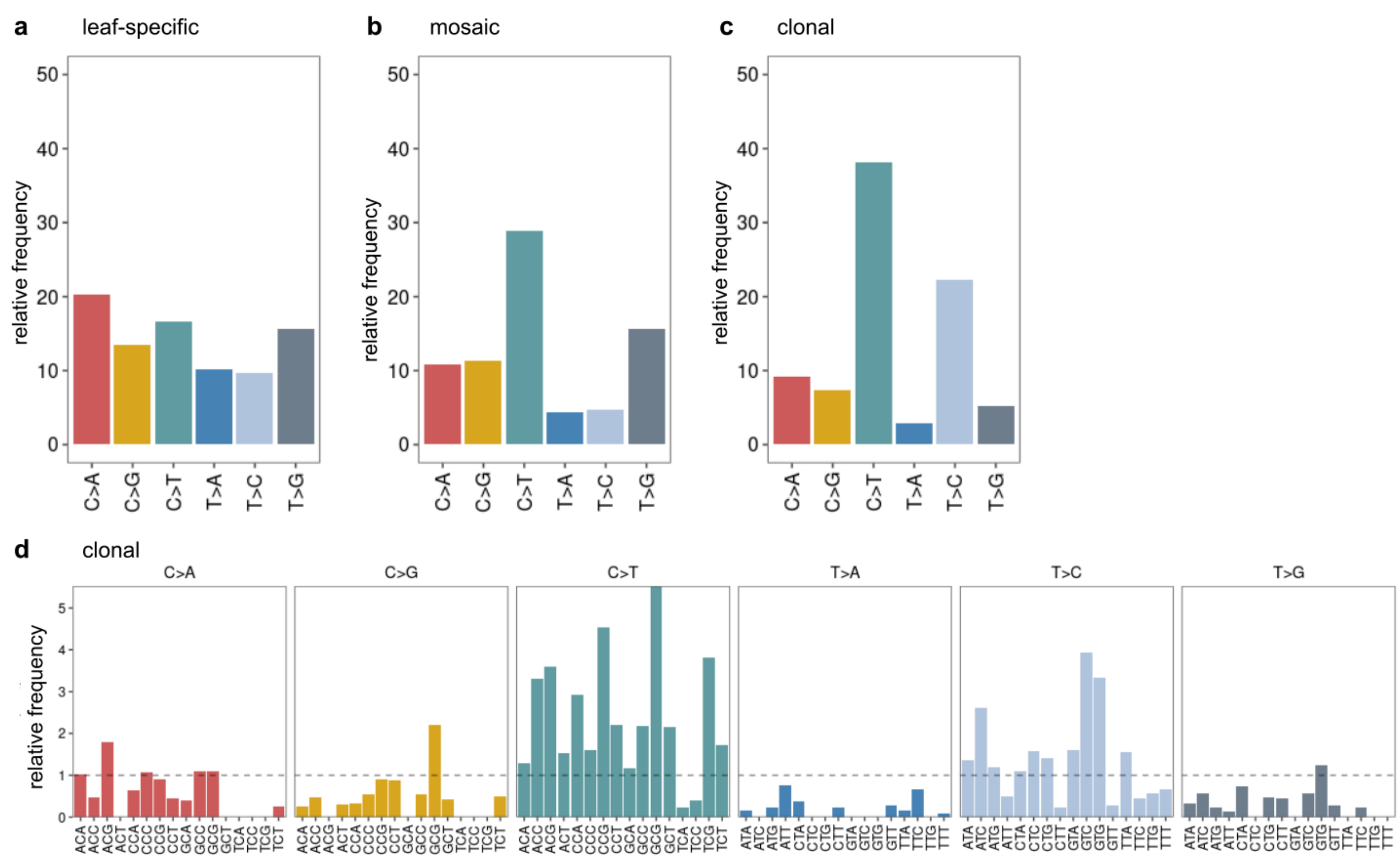

Figure S11. Mutation spectra of leaf-specific, mosaic, and clonal somatic mutations. **(a-c)** The sum of the proportion of each mutation class relative to the genomic trimer frequency for **(a)** leaf-specific, **(b)** mosaic, and **(c)** clonal somatic mutations in the historic tree. **(d)** The proportion

of each mutation class relative to the genomic trimer frequency for the set of clonal somatic mutations for the set of clonal mutations identified in the historic tree (n=218).

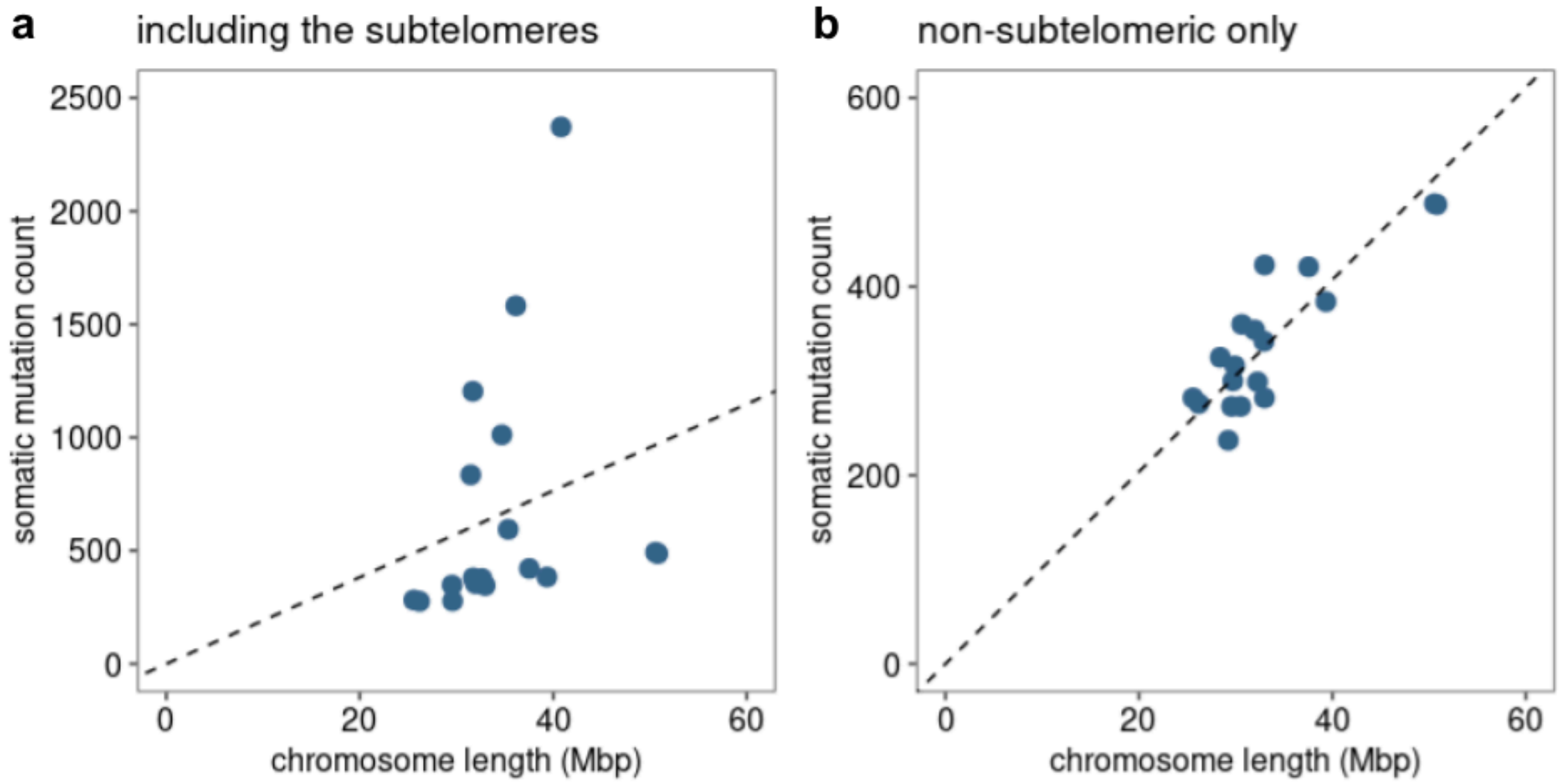

Figure S12. The relationship between somatic mutation count and chromosome length. **(a)** The number of somatic mutations detected per chromosome is not proportional to chromosome length. **(b)** The number of somatic mutations detected per chromosome is proportional to chromosome length when masking the subtelomeres.

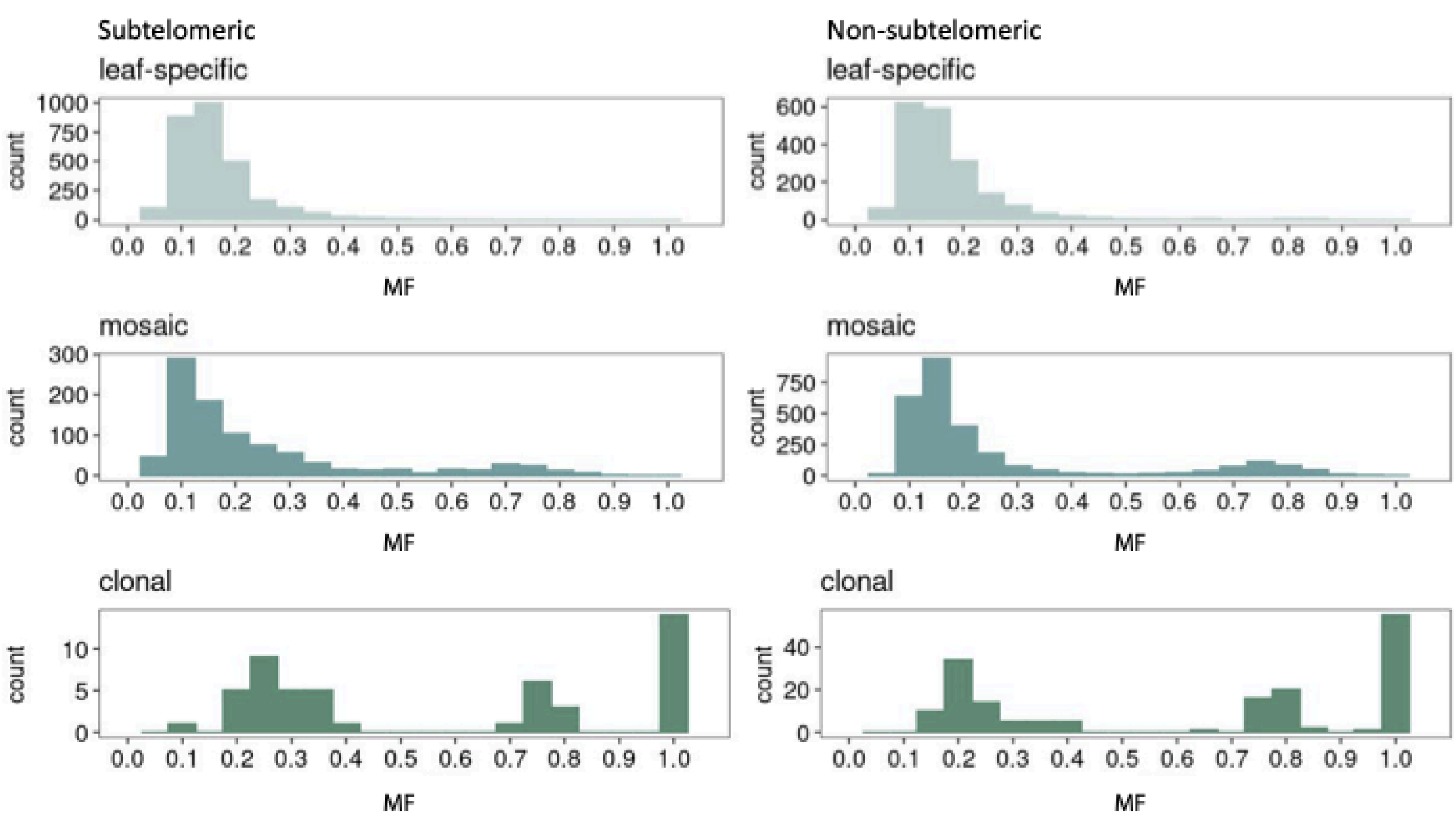

Figure S13. The distribution of mutation fraction for leaf-specific, mosaic, and clonal mutations. Similar to Fig 2, but separated into subtelomeric mutations on the left and nonsubtelomeric mutations on the right.

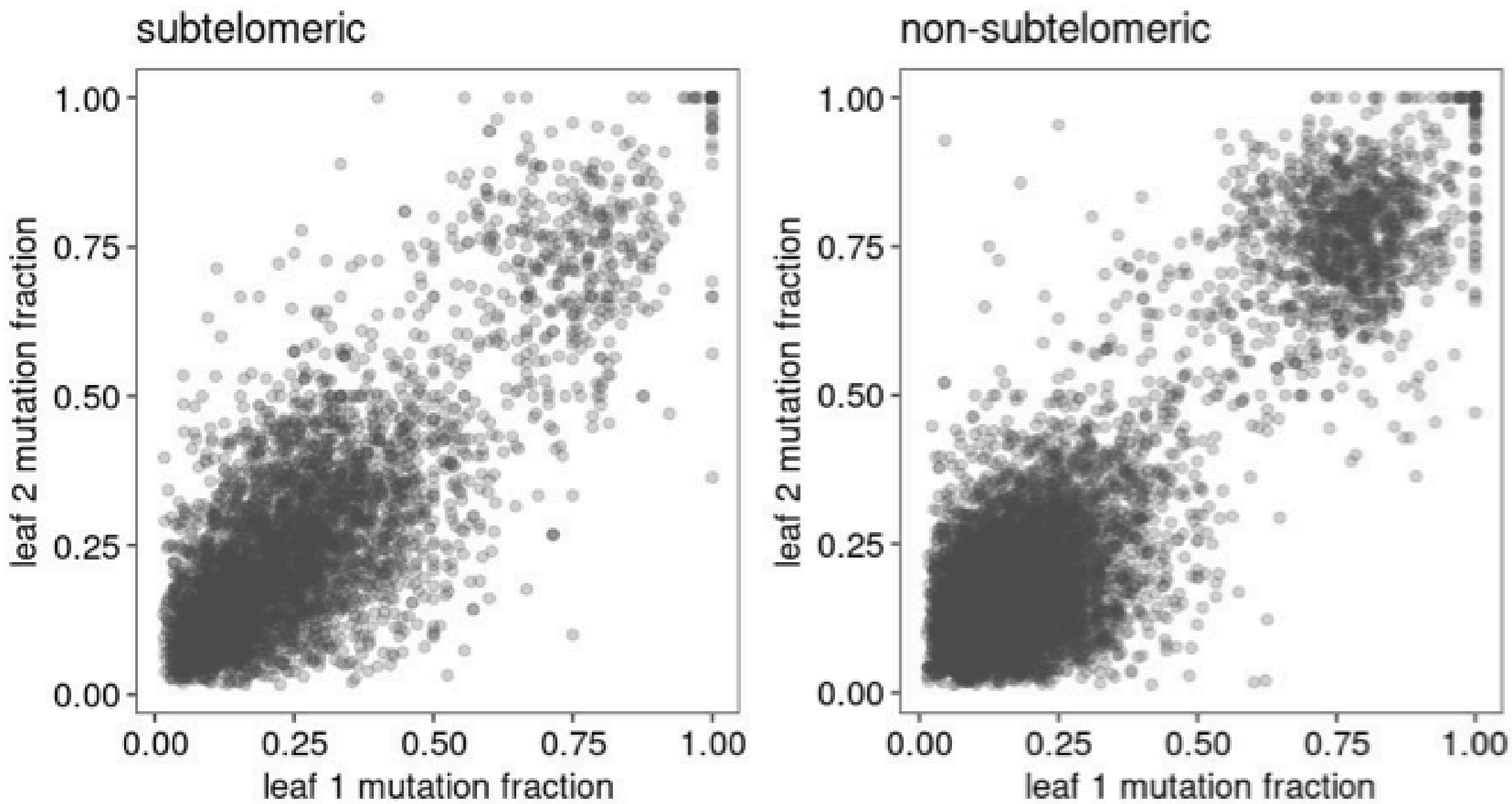

Figure S14. Comparison of the mutation fraction of somatic mutations identified in adjacent leaves. Similar to S5 but separated into subtelomeric mutations shown on the left (N=5903) and non-subtelomeric on the right (N=6122).

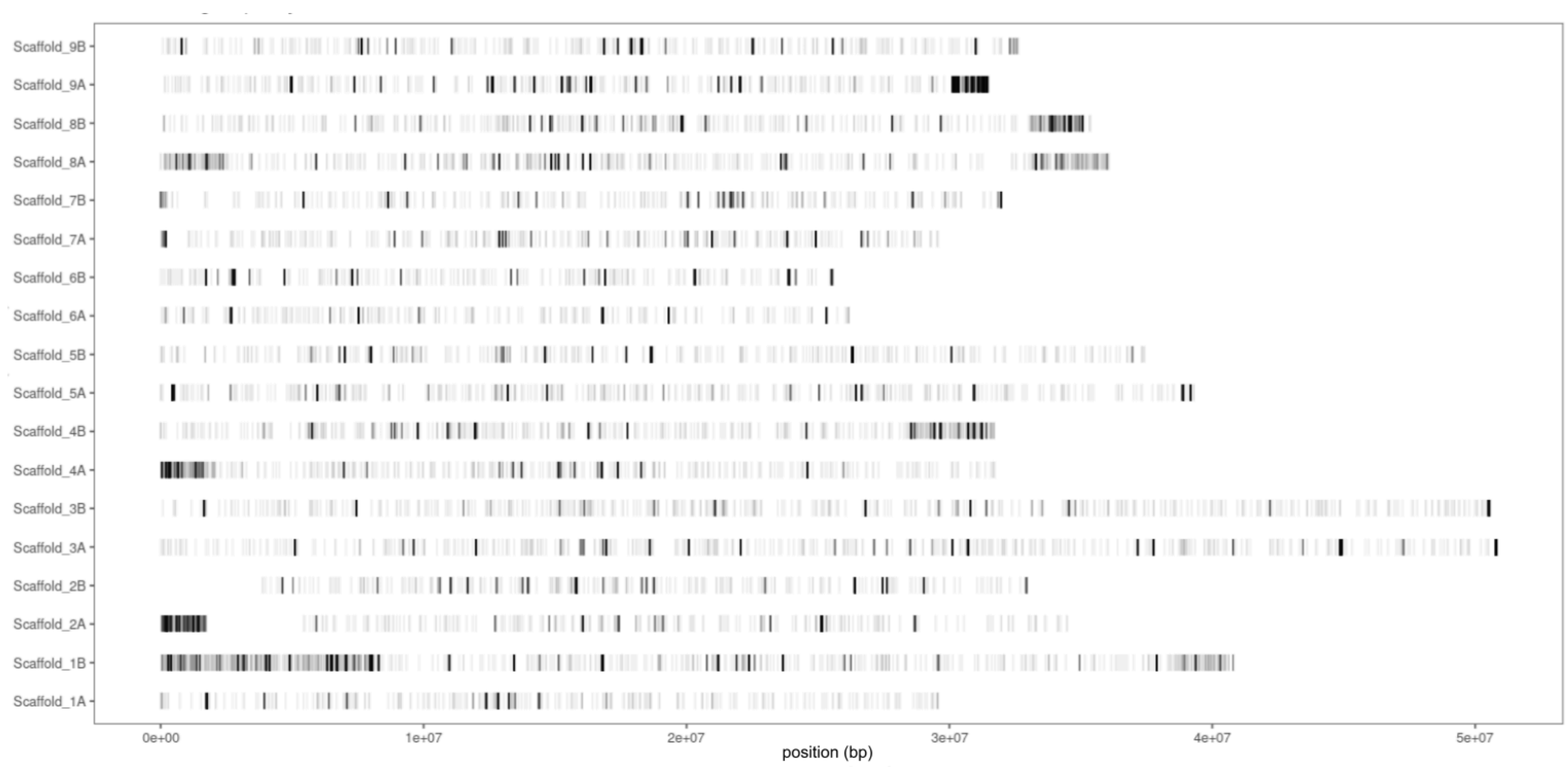

Figure S15. Chromosomal distribution of SNVs detected in 6 sweet orange individuals using PacBio Hifi sequencing data.

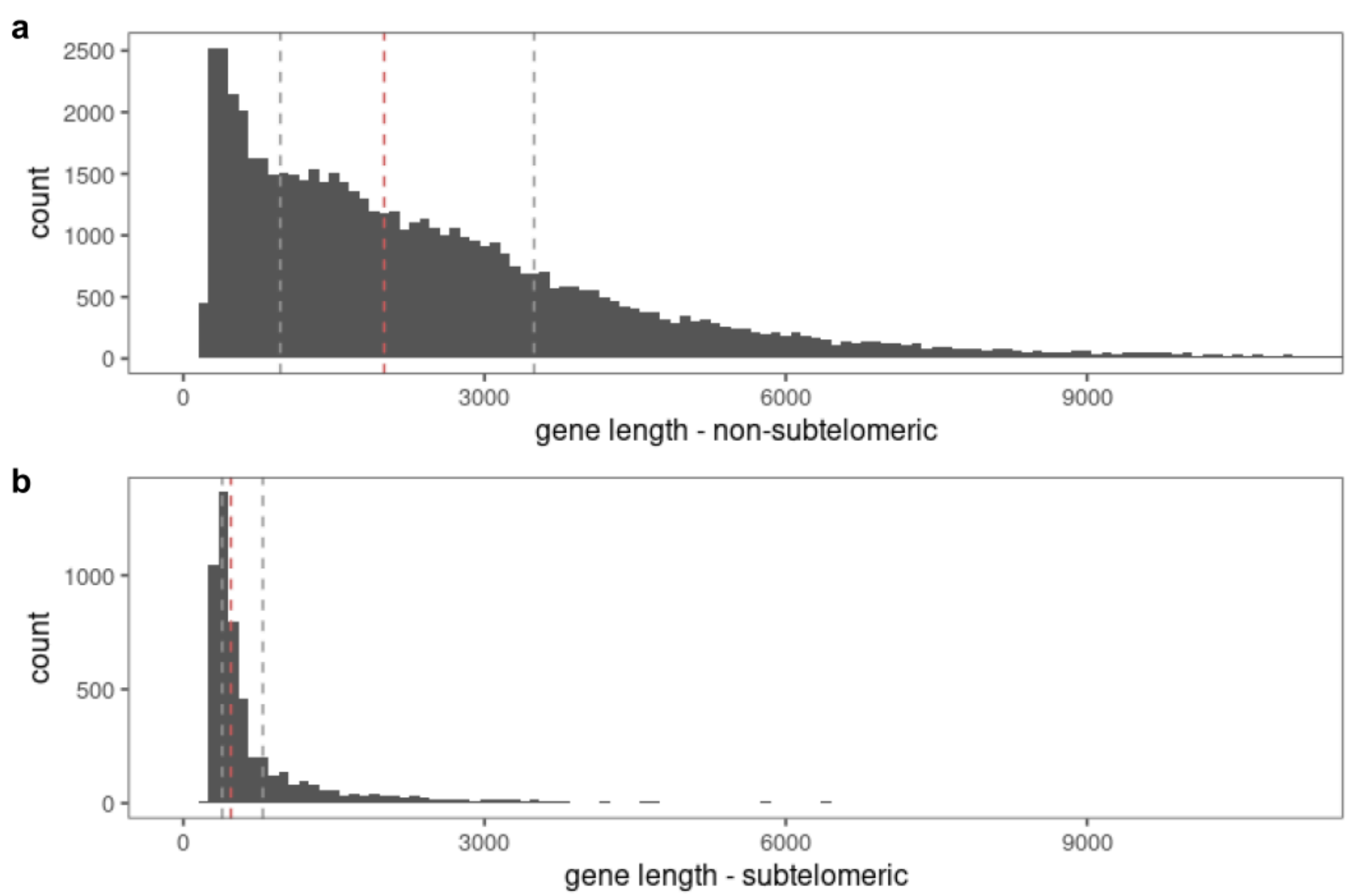

Figure S16. (a-b) Distribution of annotated gene length **(a)** in non-subtelomeric and **(b)** in subtelomeric regions. Red dashed lines indicate the mean gene length. Gray dashed lines indicate the first and third quartile

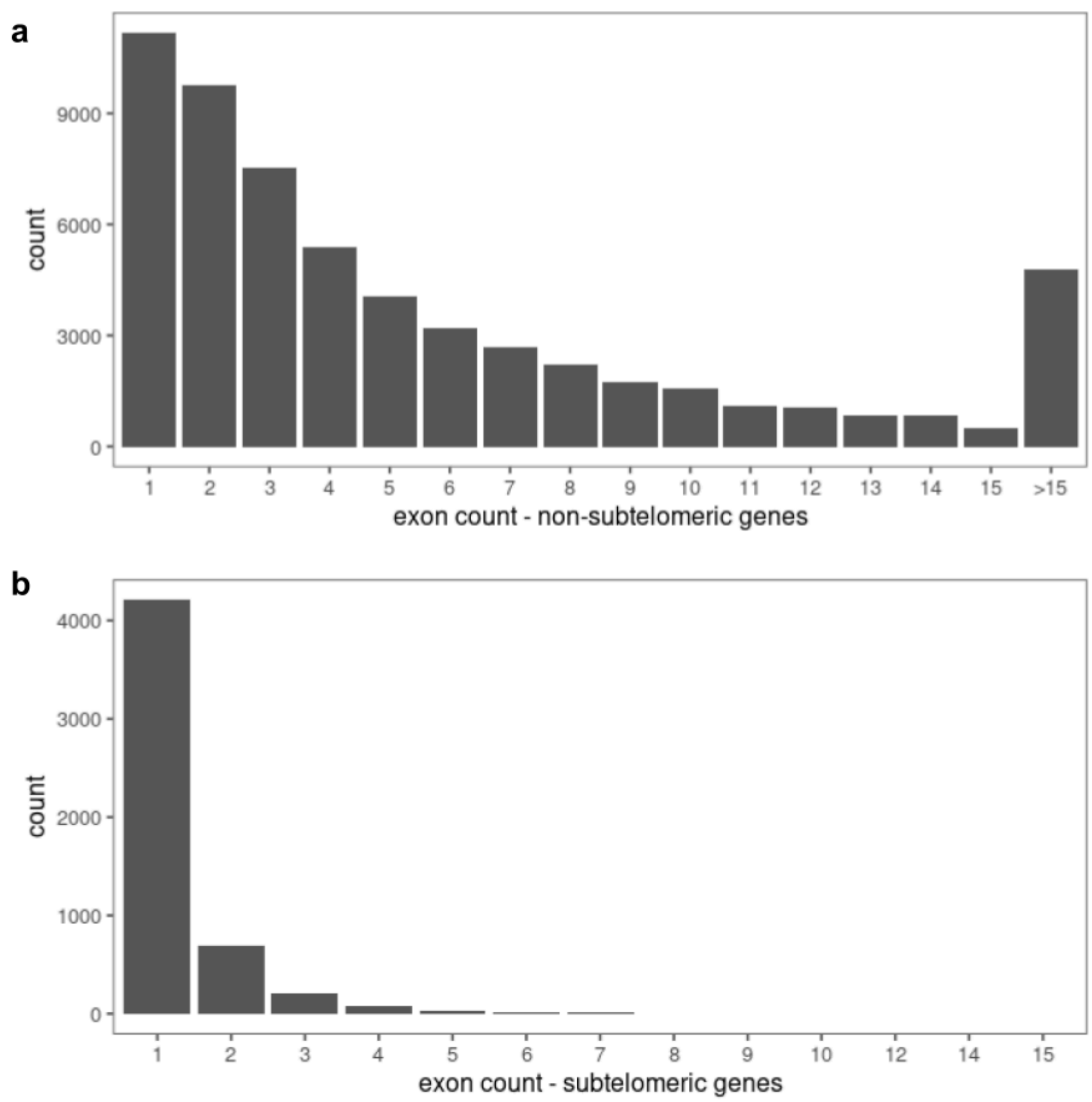

Figure S17. Number of exons in genes annotated **(a)** in the non-subtelomeric and **(b)** subtelomeric regions.

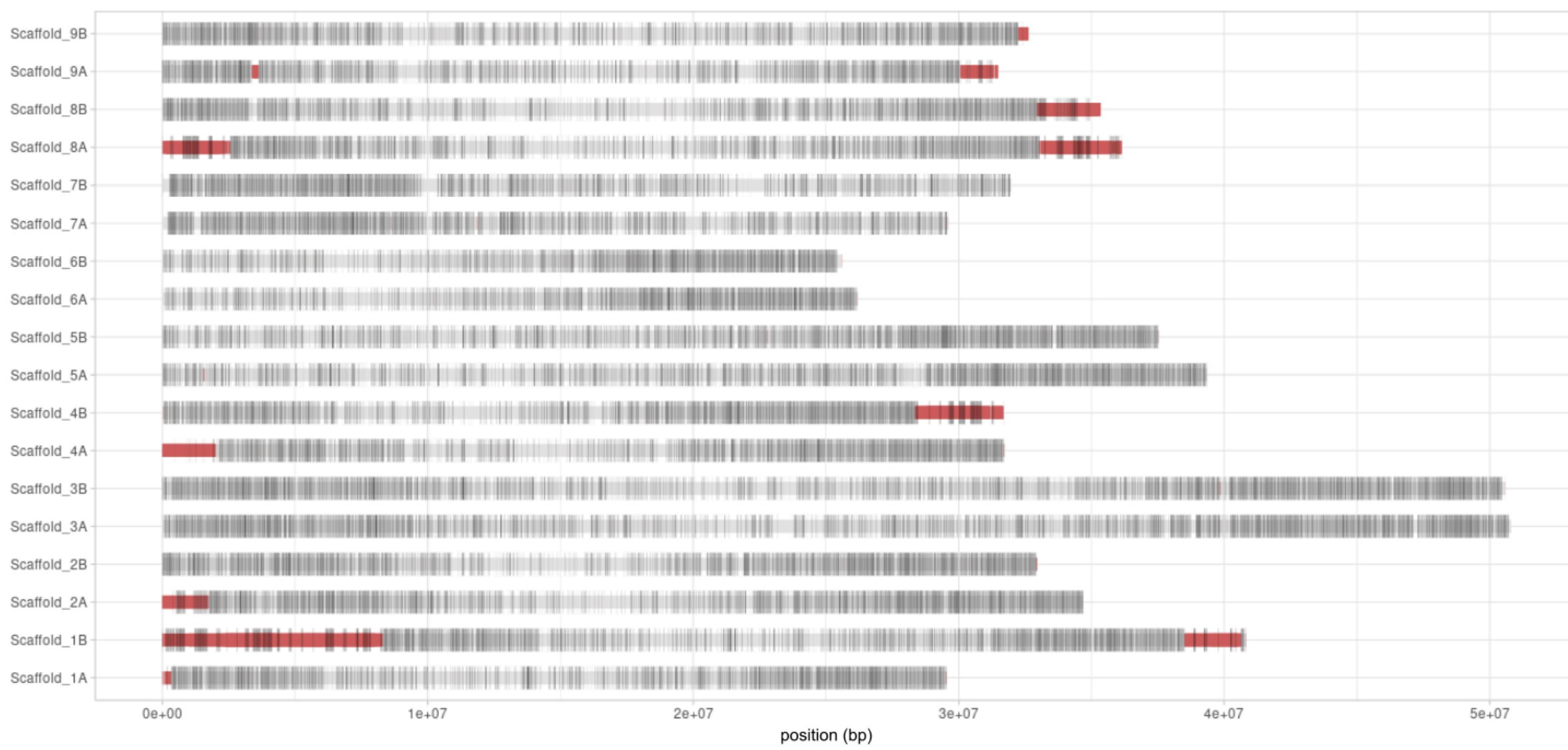

Figure S18. Genomic distribution of annotated protein-coding genes in the 'Washington navel' reference assembly. Subtelomeric regions are shown in red.

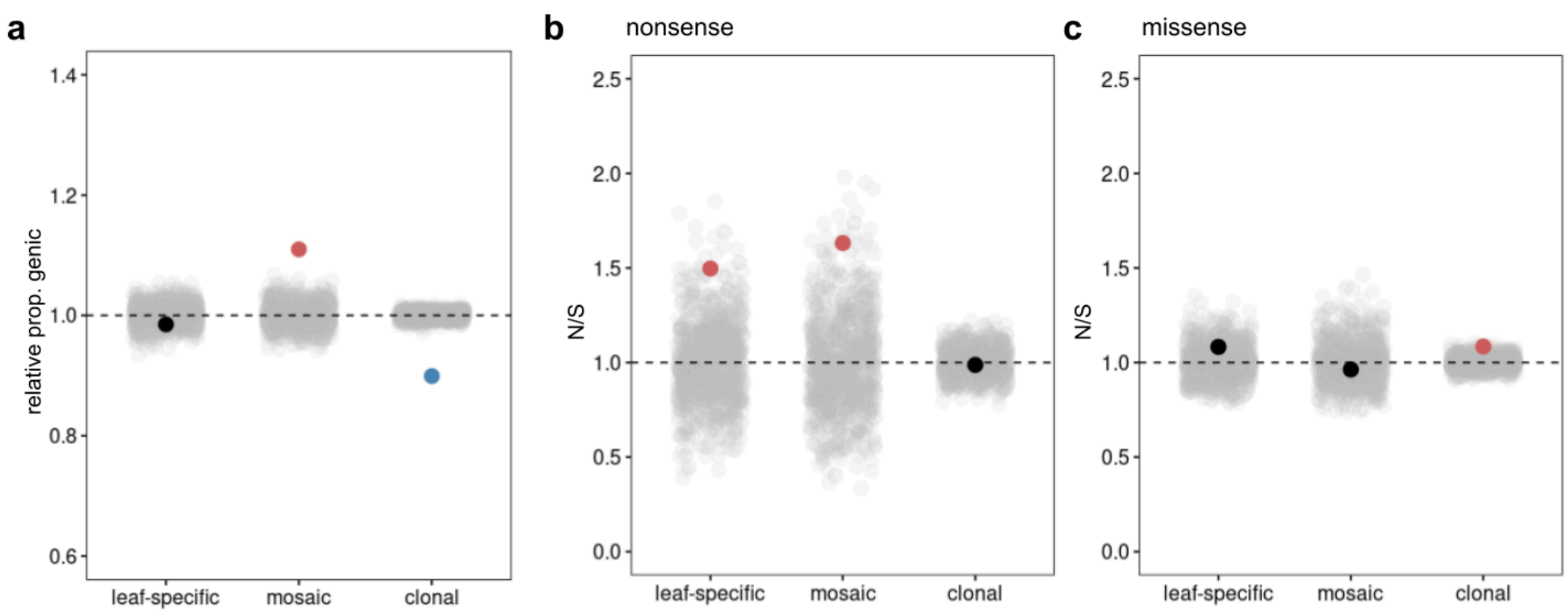

Figure S19. Proportion of genic mutations and dN/dS in the subtelomeres. Only mutations residing in subtelomeric regions were considered ($n_{leaf\text{-}specific}$ = 2888, n_{mosaic} = 935, n_{clonal} = 50). **(a)** The proportion of somatic mutations in genic regions relative to the genomic expectation based on permutation. **(b)** N/S calculated as the ratio of nonsense to synonymous mutations relative to the genomic expectation. **(c)** N/S calculated as the ratio of missense to synonymous mutations relative to the genomic expectation. Points are colored by whether they are significantly different from the genomic expectation (red = enriched, blue = depleted) or not (black). The genomic expectation is calculated as the mean value from 1000 permutations of randomly introduced *in silico* mutations (gray points) of the same sample size and mutation spectrum as the mutation set.

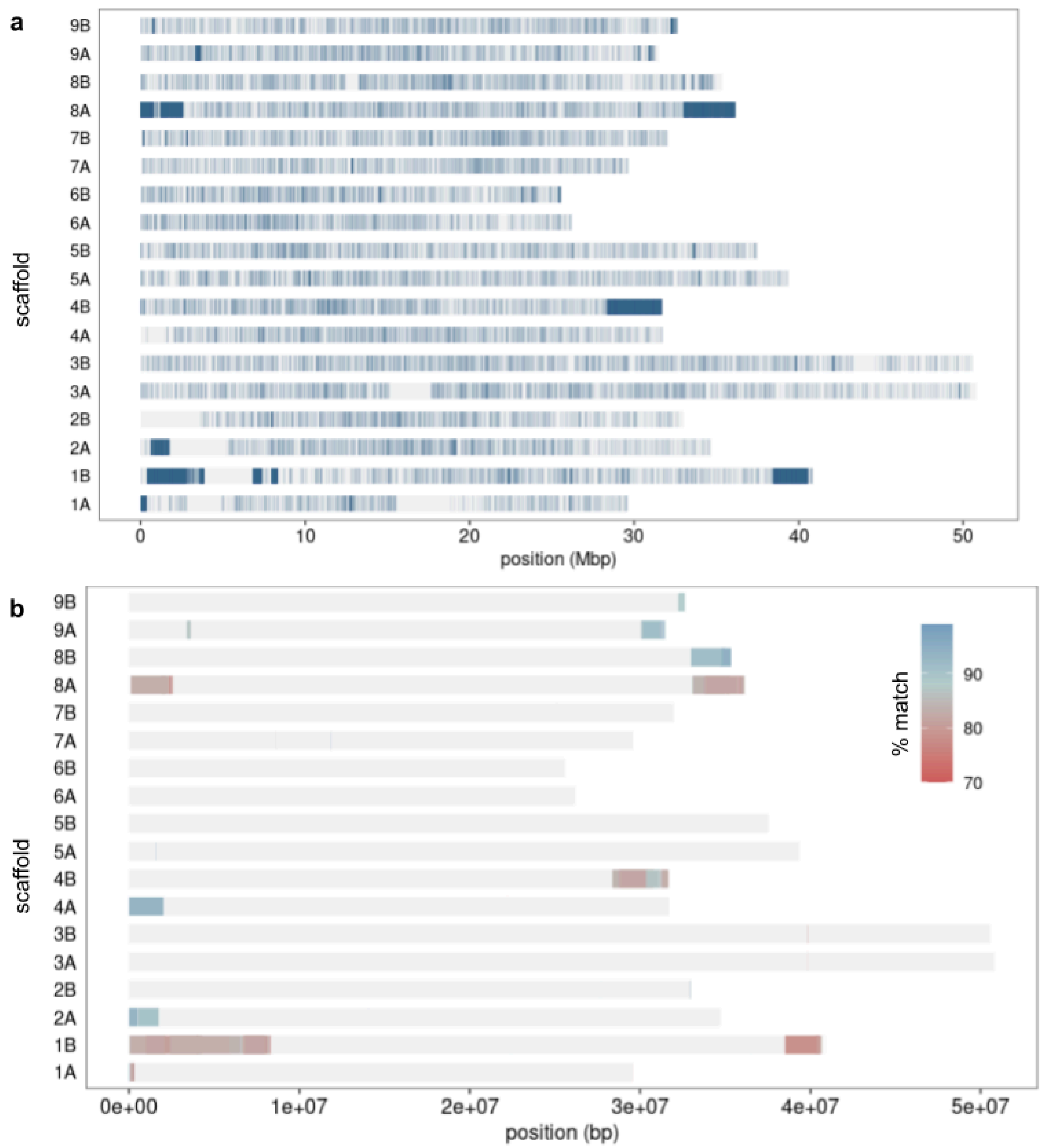

Figure S20. Clonal mutation accumulation in the subtelomeres. **(a)** The chromosomal distribution of SNVs detected in 199 clonally related accessions of sweet orange sequenced using Illumina short reads. **(b)** Subtelomeric regions colored by percent matches (percent of matches between adjacent copies overall).

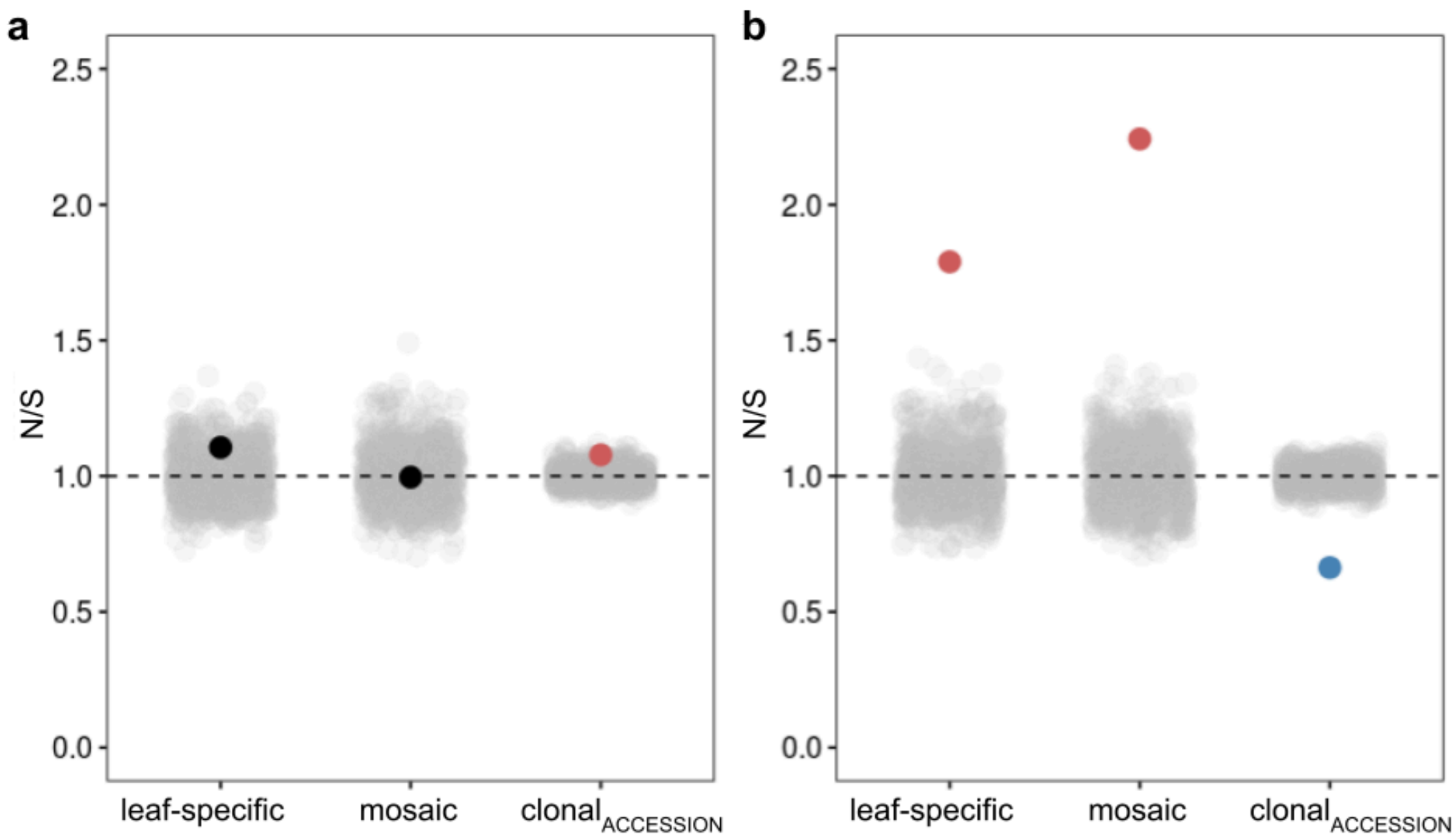

Figure S21. Combined N/S calculated as the ratio of nonsynonymous (nonsense + missense) mutations to synonymous mutations for the set of leaf-specific, mosaic, and $\text{clonal}_{\text{ACCESSION}}$ mutations **(a)** within the subtelomeres, and **(b)** outside of the subtelomeres. Points are colored if they are significantly different from the genomic expectation (red = significantly greater, blue = significantly lower) or black if they are not significantly different. The genomic expectation is calculated as the mean value from 1000 sets of randomly introduced *in silico* mutations with the same sample size and mutation spectrum as the mutation set (shown in gray).

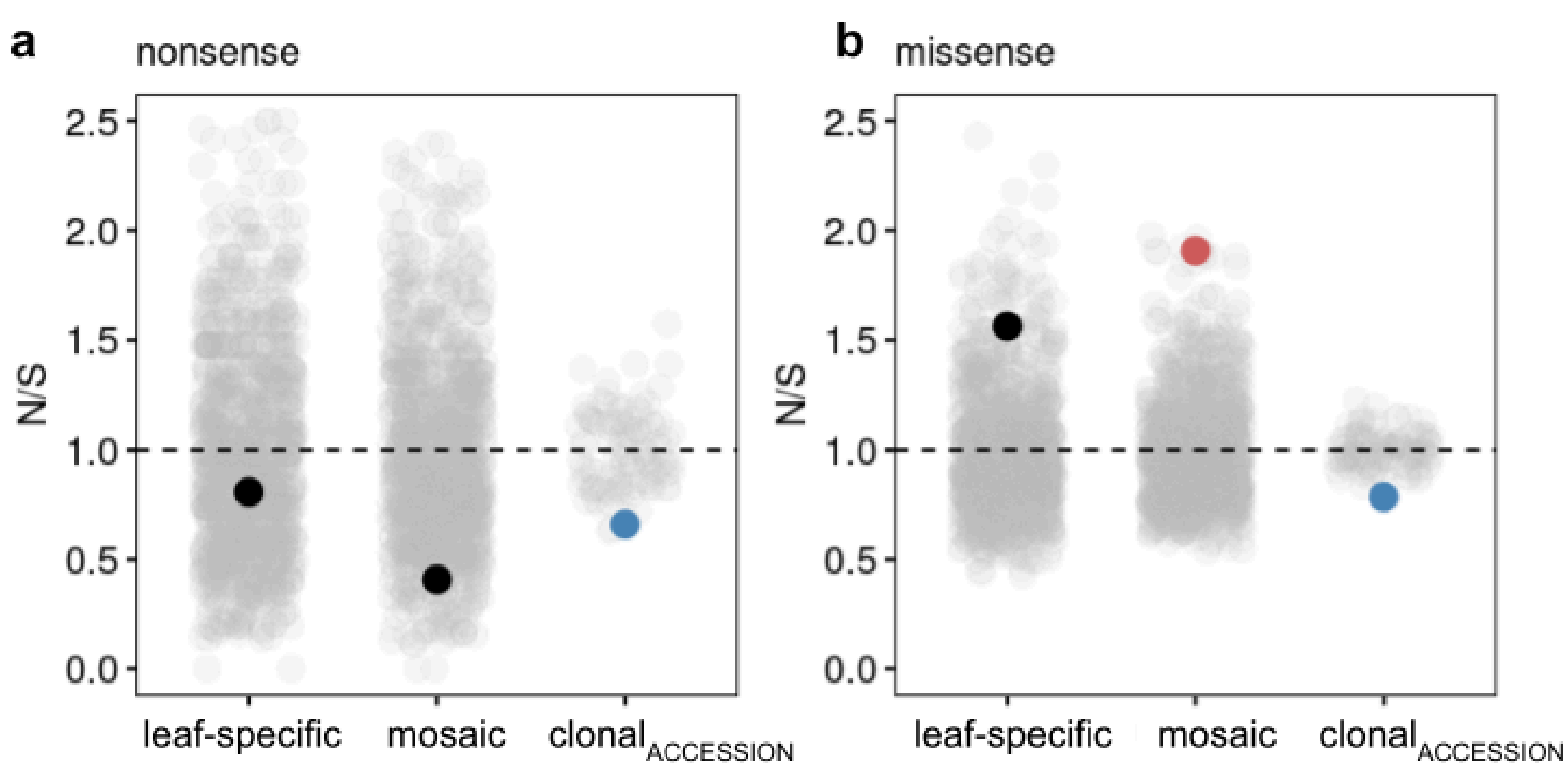

Figure S22. The ratio of N/S excluding mutations in repetitive sequences. Only mutations residing in non-subtelomeric regions that did not intersect repetitive elements were considered ($n_{leaf\text{-}specific}$ = 809, n_{mosaic} = 1063, n_{clonal} = 7195). **(a)** N/S calculated as the ratio of nonsense to synonymous mutations relative to the genomic expectation. **(c)** N/S calculated as the ratio of missense to synonymous mutations relative to the genomic expectation. Points are colored by whether they are significantly different from the genomic expectation (red = enriched, blue = depleted) or not (black). The genomic expectation is calculated as the mean value from 1000 permutations of randomly introduced *in silico* mutations (gray points) of the same sample size and mutation spectrum as the mutation set.

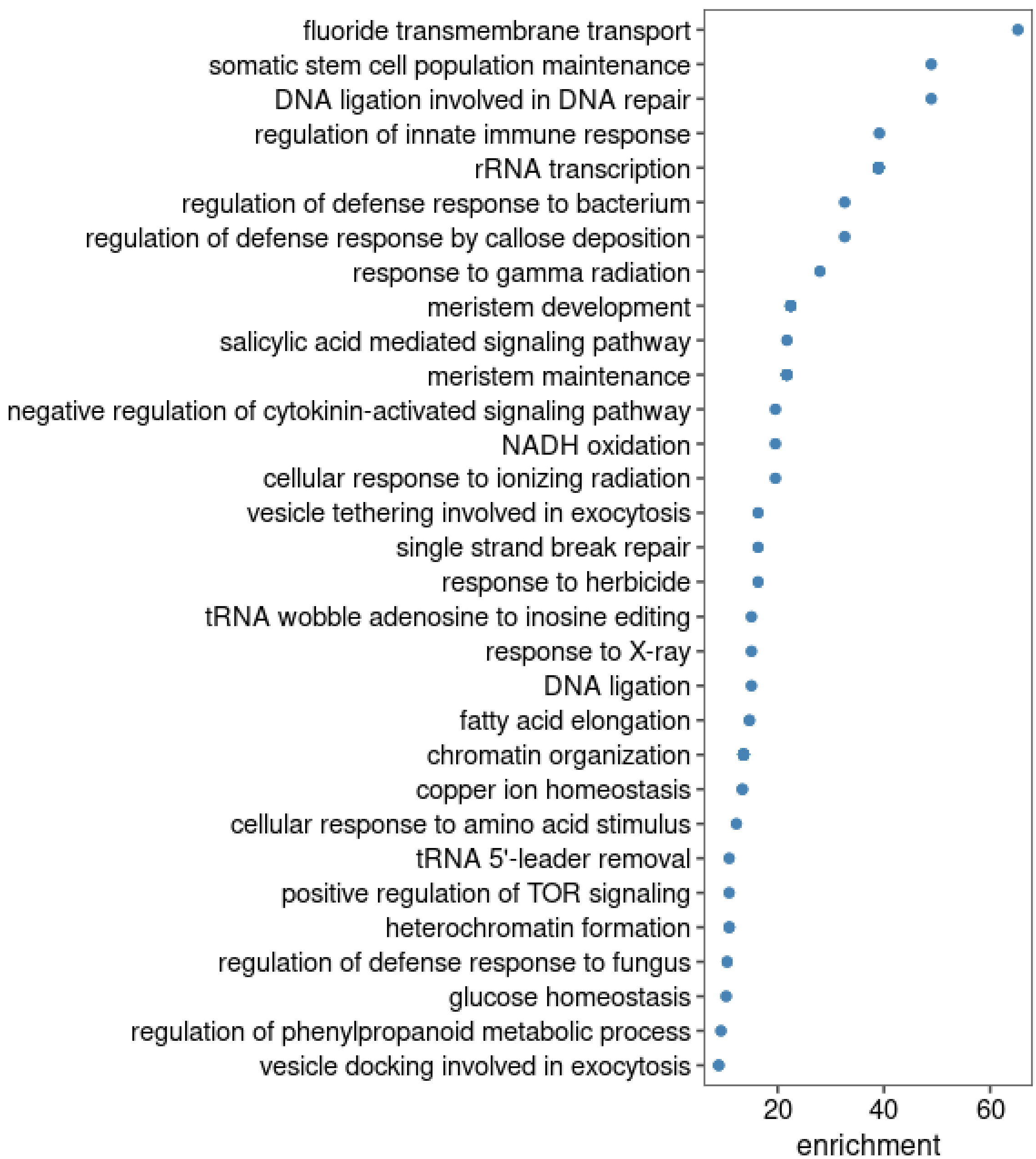

Figure S23. Biological process terms in the top 20% of over-represented GO terms among genes containing at least two missense mutations.

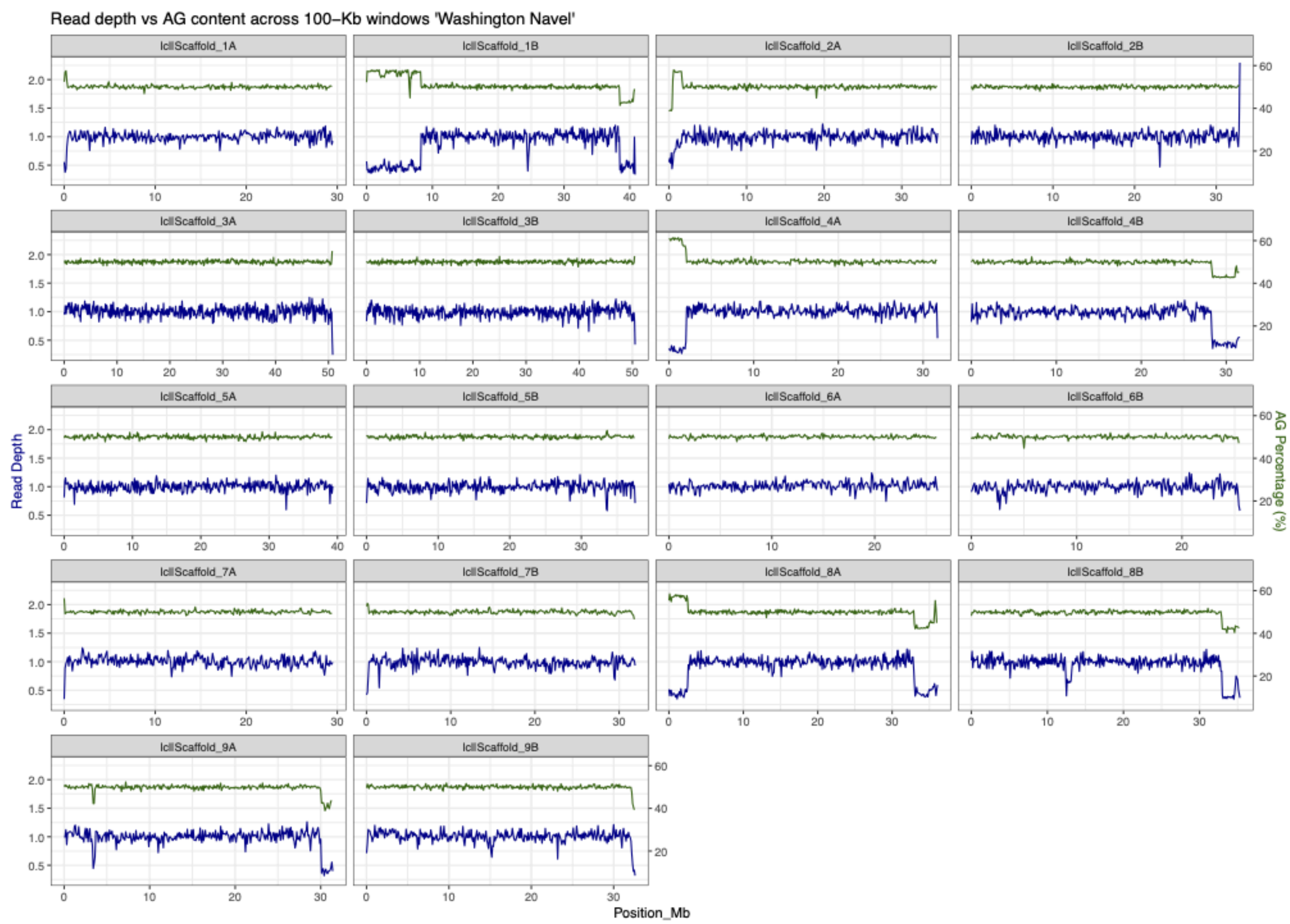

Figure S24. Normalized PacBio HiFi sequence read depth relative to AG base content across the 'Washington navel' genome. Dark blue lines correspond to the normalized PacBio read depth in 100-kb windows relative to the average genome-wide depth. Dark green lines correspond to the percentage of AG base content across the same 100-Kb windows.

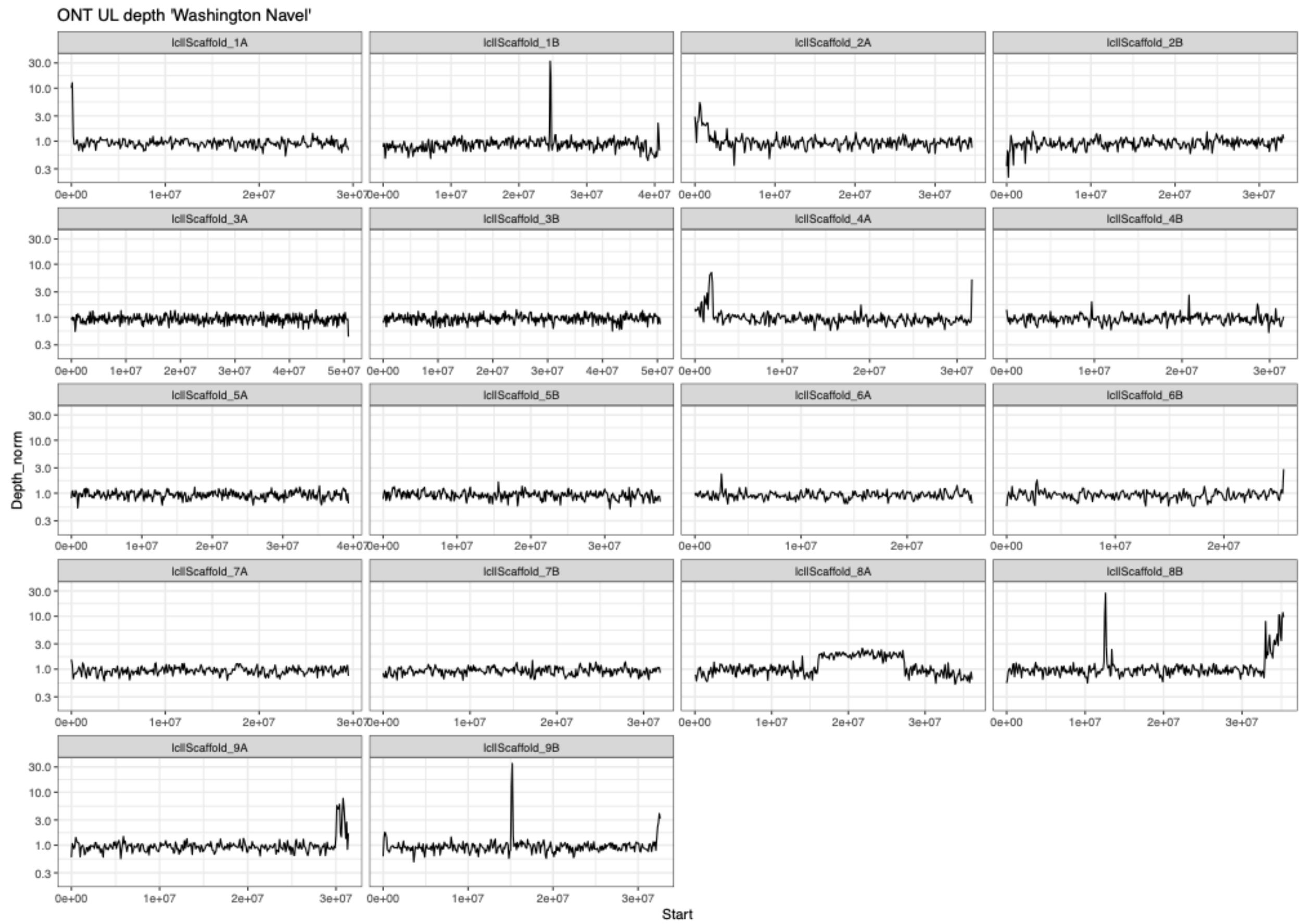

Figure S25. Normalized ONT ultra-long read depth across the 'Washington navel' genome. Black lines correspond to the normalized ONT read depth in 100-kb windows relative to the average genome-wide depth.

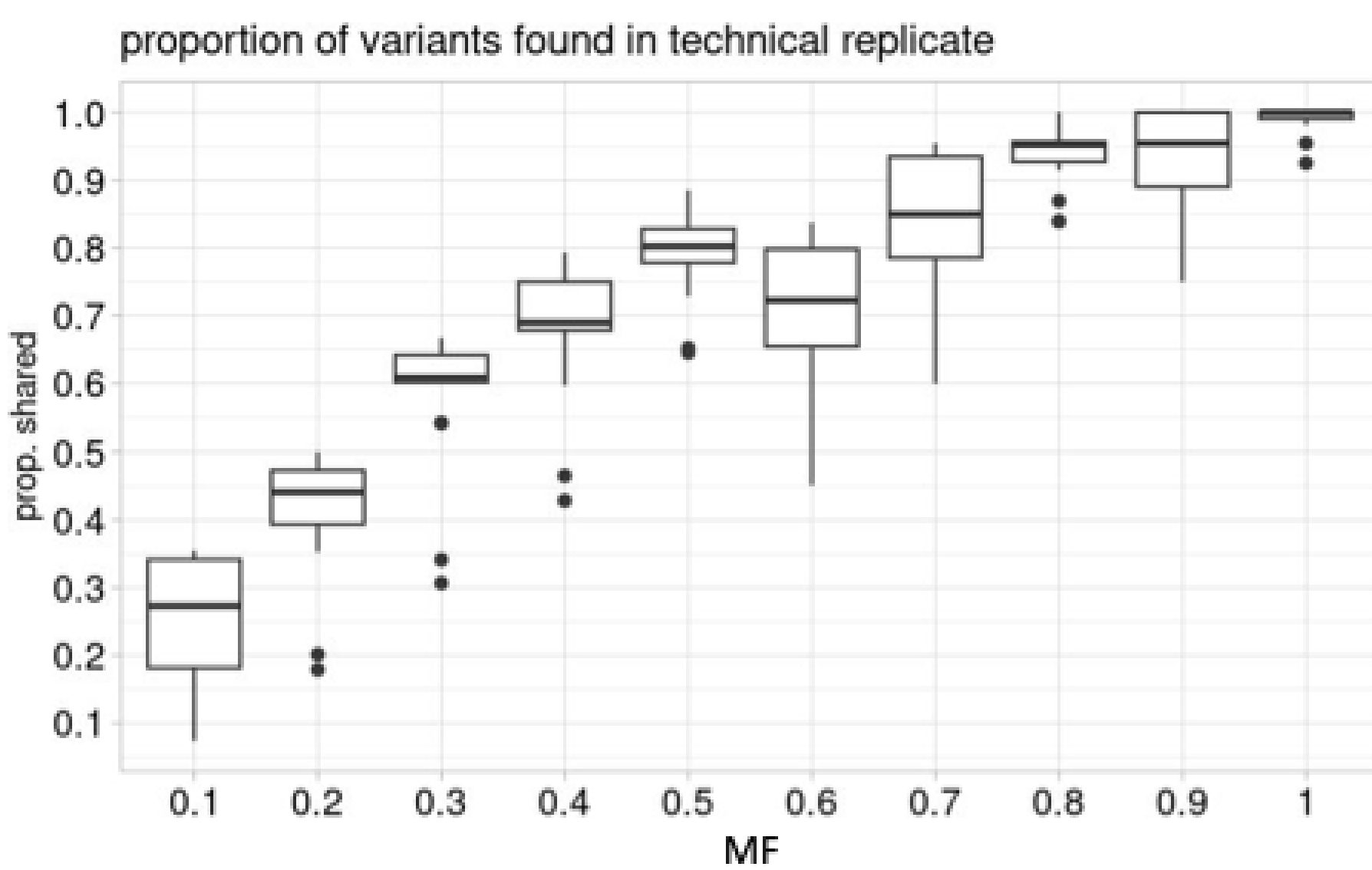

Figure S26. Concordance between variant calls across 2 technical replicates for 6 samples. The proportion of variants identified in both technical replicates across the mutation fraction spectrum.

Tables

Table S1. Sequencing & read mapping metrics. * Depth = per-haplotype average sequencing depth.

Sample ID	Branch	Location	Leaf	Mutation count	Depth*	NCBI accession
1A_1	1	apical	1	2026	42.66	SAMN42644081
1A_2	1	apical	2	3407	42.21	SAMN42644082
1B_1	1	basal	1	2713	33.89	SAMN42644083
1B_2	1	basal	2	2179	48.86	SAMN42644084
2A_1	2	apical	1	1804	39.22	SAMN42644085
2A_2	2	apical	2	2024	43.91	SAMN42644086
2B_1	2	basal	1	2028	39.03	SAMN42644087
2B_2	2	basal	2	1074	32.26	SAMN42644088
3A_1	3	apical	1	1859	41.97	SAMN42644089
3A_2	3	apical	2	3578	46.31	SAMN42644090
3B_1	3	basal	1	3157	46.71	SAMN42644091
3B_2	3	basal	2	1961	45.15	SAMN42644092
4A_1	4	apical	1	1813	37.38	SAMN42644093
4A_2	4	apical	2	1543	37.35	SAMN42644094
4B_1	4	basal	1	2282	60.83	SAMN42644095
4B_2	4	basal	2	1446	35.05	SAMN42644096
5A_1	5	apical	1	1340	32.89	SAMN42644097
5A_2	5	apical	2	1617	34.83	SAMN42644098
5B_1	5	basal	1	1705	35.97	SAMN42644099
5B_2	5	basal	2	3904	46.13	SAMN42644100
6A_1	6	apical	1	1935	43.01	SAMN42644101
6A_2	6	apical	2	1994	42.05	SAMN42644102
6B_1	6	basal	1	897	16.43	SAMN42644103
6B_2	6	basal	2	1947	41.59	SAMN42644104
7A_1	7	apical	1	2073	43.17	SAMN42644105
7A_2	7	apical	2	2578	36.79	SAMN42644106

7B_1	7	basal	1	1926	46.08	SAMN42644107
7B_2	7	basal	2	1994	43.84	SAMN42644108

Table S2. Detection rates of *in silico* mutations introduced into 40X simulated sequencing data. Simulated sequencing reads were generated from a single chromosome pair of the diploid reference genome. * ploidy = GATK HaplotypeCaller ploidy parameter

ploidy*		introduced mutation fraction									
		0.1	0.2	0.3	0.4	0.5	0.6	0.7	0.8	0.9	1
recall (%)	1	0.1	0.3	0.8	2.8	16.5	84.0	92.2	93.5	93.6	77.9
	2	11.0	74.1	92.3	93.8	95.0	95.2	95.6	95.1	94.4	78.8
	4	69.4	87.8	93.3	94.1	95.2	95.4	95.7	95.2	94.5	78.8
	10	78.0	89.6	93.5	93.9	94.7	94.8	95.0	94.8	94.3	78.2
specificity (%)	1	100.0	100.0	100.0	100.0	100.0	100.0	100.0	100.0	100.0	100.0
	2	100.0	99.9	99.9	99.9	100.0	100.0	99.9	100.0	100.0	100.0
	4	97.5	98.8	99.7	99.8	100.0	100.0	99.9	100.0	100.0	100.0
	10	70.2	98.3	99.3	99.7	100.0	100.0	99.9	100.0	100.0	100.0

Table S3. Sequencing depth for 2 technical replicates for 6 samples from the historic tree.
*per-haplotype average sequencing depth based on aligned reads

Sample ID	Branch	Location	Leaf	Replicate	Depth*
1_6A1	6	apical	1	1	65.8
2_6A1	6	apical	1	2	65.3
1_6A2	6	apical	2	1	63.2
2_6A2	6	apical	2	2	22.6
1_6B1	6	basal	1	1	22.9
2_6B1	6	basal	1	2	58.0
1_6B2	6	basal	2	1	82.3
2_6B2	6	basal	2	2	54.0
1_7A1	7	apical	1	1	73.4
2_7A1	7	apical	1	2	64.3

1_7B1	7	basal	2	1	89.0
2_7B1	7	basal	2	2	86.8

Table S4. Percent of mutations identified in the original sample that were also identified in each technical replicate. Rows are the original sample, columns are the technical replicates. Clonal, mosaic, and leaf-specific mutations are shown separately.

Clonal:

	6A1		6A2		6B1		6B2		7A1		7B1	
	1	2	1	2	1	2	1	2	1	2	1	2
6A	**100**	**100**	**100**	**95**	94	99	100	100	100	100	100	100
6B	100	100	100	95	**94**	**99**	**100**	**100**	100	100	100	100
7A	100	100	100	95	94	99	100	100	**100**	**100**	100	100
7B	100	100	100	95	94	99	100	100	100	100	**100**	**100**

Mosaic:

	6A1		6A2		6B1		6B2		7A1		7B1	
	1	2	1	2	1	2	1	2	1	2	1	2
6A	**99**	**100**	**100**	**89**	12	12	12	12	0	0	0	0
6B	27	27	32	19	**94**	**100**	**100**	**100**	0	0	0	0
7A	0	0	0	0	1	0	1	0	**99**	**100**	21	21
7B	0	0	0	0	0	0	0	0	30	30	**98**	**98**

Leaf-specific:

	6A1		6A2		6B1		6B2		7A1		7B1	
	1	2	1	2	1	2	1	2	1	2	1	2
6A	**47**	**47**	**60**	**51**	0	0	0	0	2	0	0	0
6B	0	0	0	0	**40**	**67**	**53**	**67**	0	0	0	0
7A	17	21	17	12	0	17	21	17	**54**	**62**	8	8
7B	0	8	8	8	8	15	8	8	0	15	**62**	**54**

Table S5. Percent of mutations identified in the original sample that were also identified in either one or both of the technical replicates. Rows are the original sample, columns are the combined technical replicates. Clonal, mosaic, and leaf-specific mutations are shown separately.

Clonal:

	6A1	6A2	6B1	6B2	7A1	7B1
6A	**100**	**100**	100	100	100	100
6B	100	100	**100**	**100**	100	100
7A	100	100	100	100	**100**	100
7B	100	100	100	100	100	**100**

Mosaic:

	6A1	6A2	6B1	6B2	7A1	7B1
6A	**100**	**100**	12	12	0	0
6B	27	32	**100**	**100**	0	0
7A	1	0	1	1	**100**	21
7B	0	0	0	0	30	**100**

Leaf-specific:

	6A1	6A2	6B1	6B2	7A1	7B1
6A	**49**	**60**	0	0	2	0
6B	0	0	**67**	**67**	0	0
7A	33	29	17	33	**67**	17
7B	8	8	15	15	15	**69**

Table S6. Mutation density (number of mutations detected per Mbp) in subtelomeric and non-subtelomeric regions.

	mutation density (per Mbp)	
	subtelomeric	non-subtelomeric
leaf-specific	104.8	3.2
mosaic	33.9	4.5
Illumina $\text{clonal}_{\text{HISTORIC}}$	1.8	0.3
Illumina $\text{clonal}_{\text{ACCESSION}}$	323.8	31.8
PacBio HiFi $\text{clonal}_{\text{ACCESSION}}$	176.5	34.8

Table S7. PacBio HiFi sequencing & read mapping metrics for samples collected from six sweet orange varieties. CRC number used to identify each accession in the Givaudan Citrus Variety Collection at the University of California, Riverside. Depth is calculated as per-haplotype average sequencing depth from reads mapped to the 18 primary scaffolds. PacBio sequencing reads are available under NCBI BioProject PRJNA1272085.

Sample ID	CRC	Variety Name	Depth
PWN	1241	Parent Washington Navel	40.4
OLI	2750	Olinda Valencia	35.5
MOR	3830	Moro Blood orange	41.9
POW	4040	Powell Navel orange	41.9
CAR	3994	Cara Cara Navel orange	42.3
SHA	4258	Shahani Red Navel	37.7

Table S8. Proportion of mutations in annotated gene regions ($prop_{mut}$) excluding the subtelomeres. The genomic expectation ($prop_{exp}$) is calculated as the mean of 1000 permutations. For $prop_{mut}$ values outside of a 95% CI, the p-value is the likelihood of a permutation value at least as extreme as the mutation value.

	n	n_{genic}	$prop_{mut}$	$prop_{exp}$	p-value
leaf-specific	1906	1259	0.661	0.682	0.001
mosaic	2725	1748	0.641	0.682	0.001
$clonal_{HISTORIC}$	168	93	0.554	0.680	0.001
$clonal_{ACCESSION}$	19085	10294	0.539	0.680	0.001

Table S9. The ratio of nonsynonymous to synonymous mutation (N/S_{mut}) excluding the subtelomeres. The genomic expectation (N/S_{exp}) is calculated from 1000 permutations. For N/S_{mut} values outside of a 95% CI of N/s_{exp}, the p-value is the likelihood of a permutation value at least as extreme as the mutation value.

		n	N_{mut}	S_{mut}	N/S_{mut}	N/S_{exp}	p-value
nonsense	leaf-specific	1906	6	24	0.250	0.2560	--
	mosaic	2725	3	32	0.094	0.216	0.005
	$clonal_{HISTORIC}$	168	0	4	0	0.051	--
	$clonal_{ACCESSION}$	19085	51	435	0.117	0.211	0.001
missense	leaf-specific	1906	162	24	6.750	3.627	0.002
	mosaic	2725	259	32	8.094	3.428	0.002
	$clonal_{HISTORIC}$	168	6	4	1.500	3.840	--
	$clonal_{ACCESSION}$	19085	792	435	1.821	2.710	0.001
total	leaf-specific	1906	168	24	7.000	3.912	0.002
	mosaic	2725	262	32	8.188	3.652	0.002
	$clonal_{HISTORIC}$	168	6	4	1.500	4.318	--
	$clonal_{ACCESSION}$	19085	843	435	1.938	2.924	0.001

Table S10. The total number and proportion of each category of somatic mutation that intersect the repeat annotation of 'Washington navel'.

	total		outside the subtelomeres only	
	count	proportion	count	proportion
leaf-specific	4077	0.85	1188	0.62
mosaic	2778	0.76	1843	0.68
clonal	166	0.76	116	0.69

Table S11. The ratio of nonsynonymous to synonymous mutation (N/S_{mut}) excluding the subtelomeres and excluding mutations intersecting the repeat annotation. The genomic expectation (N/S_{exp}) is calculated from 1000 permutations. The p-value is the likelihood of a permutation value at least as extreme as the mutation value.

		n	N_{mut}	S_{mut}	N/S_{mut}	N/S_{exp}	p-value
nonsense	leaf-specific	809	6	22	0.273	0.339	0.397
	mosaic	1063	3	30	0.100	0.246	0.084
	$clonal_{ACCESSION}$	7195	58	431	0.135	0.204	0.014
missense	leaf-specific	809	158	22	7.182	4.592	0.052
	mosaic	1063	236	30	7.867	4.121	0.008
	$clonal_{ACCESSION}$	7195	935	431	2.169	2.767	0.004

Table S12. Sweet orange accessions collected and sequenced from the Givaudan Citrus Variety Collection. Raw Illumina sequencing reads are available under NCBI BioProject PRJNA1272085.

Sample	Name
CRC65	Seedy Washington Navel
CRC287	Navelencia
CRC321	Imperial Variegated

CRC371	Orange of Heaven
CRC574	Smiths Early Navel
CRC588	Golden Buckeye
CRC609	McFadden Ribbed Navel
CRC950	Lima
CRC969	Thomson Navel
CRC983	Thomson Navel
CRC1045	Selecta
CRC1203	Variegated Navel
CRC1240	Fuzzy Valencia
CRC1241	Parent Washington Navel
CRC1512	Bidwells Bar
CRC2008	Carter Navel
CRC2561	Burris Blood Valencia
CRC2750	Olinda Valencia
CRC2776	Seedless Valencia
CRC2802	Argentina
CRC3014	Newhall Navel
CRC3017	Gillette
CRC3030	Cutter Valencia
CRC3032	Campbell Valencia
CRC3033	Frost Nucellar Washington Navel
CRC3152	Doble Fina
CRC3182	Trovita Strain A
CRC3242	Vaccaro
CRC3307	Dream Navel
CRC3315	Rio Grande Navel
CRC3320	Workman Navel
CRC3339	Werley Valencia
CRC3354	Dry Navel
CRC3401	Grosse Ronde
CRC3584	Sarah, Shamouti seedling
CRC3596	Tarocco #7
CRC3645	Fischer Navel o.p. seedling
CRC3650	Entre Fina
CRC3732	Cluster Navel
CRC3746	Shamouti
CRC3791	Atwood Navel, old budline
CRC3801	Vainiglia Pink-fleshed

CRC3803	Lane Late
CRC3827	Ruby
CRC3828	Sanguinelli
CRC3830	Moro
CRC3867	Rotuma Island
CRC3903	Fukumoto
CRC3955	Midknight Valencia
CRC3963	Delta Seedless Valencia
CRC3964	Palmer navel
CRC3977	Washington Sanguine
CRC3994	Cara Cara
CRC4000	Sanguina Doble Fina
CRC4001	Variegated Valencia
CRC4015	Navelina
CRC4022	Navelate
CRC4023	Robertson Navel
CRC4037	Autumn Gold
CRC4038	Barnfield Navel
CRC4039	Chislett Navel
CRC4050	Cogan Navel
CRC4058	Ricalate Navel
CRC4063	Robyn Navel
CRC4065	Ceridwen Navel
CRC4083	Wiffen Summer Navel
CRC4084	Rohde Navel
CRC4086	Cukurova Early Navel
CRC4093	Smith Red Valencia
CRC4112	Fulwood Navel
CRC4114	Delfino
CRC4128	Rio Farms Vaniglia
CRC4141	Rufert A
CRC4151	Apopka
CRC4168	Bahianinha Araras
CRC4175	Australian Navel
CRC4192	Gillemberg
CRC4193	Pollock T4
CRC4194	Secret Garden
CRC4195	Whitley
CRC4207	Variegated Cara Cara

CRC4224	Maltaise Demi Sanguine
CRC4228	Bitters (Newhall) navel
CRC4229	Chapman Valencia
CRC4232	Rocky Hill Valencia
CRC4258	Shahani Red Navel
CRC4262	Rush TI

Table S13. Proportion of mutations in annotated gene regions ($prop_{mut}$) in the subtelomeres. The genomic expectation ($prop_{exp}$) is calculated as the mean of 1000 permutations. For $prop_{mut}$ values outside of a 95% CI of $prop_{exp}$, the p-value is the likelihood of a permutation value at least as extreme as the mutation value.

	n	n_{genic}	$prop_{mut}$	$prop_{exp}$	p-value
leaf-specific	2888	1090	0.377	0.383	--
mosaic	935	398	0.426	0.383	0.001
$clonal_{HISTORIC}$	50	22	0.440	0.386	--
$clonal_{ACCESSION}$	8922	3085	0.346	0.385	0.001

Table S14. The ratio of nonsynonymous to synonymous mutation (N/S_{mut}) in the subtelomeres. The genomic expectation (N/S_{exp}) is calculated from 1000 permutations. For N/S_{mut} values outside of a 95% CI of N/s_{exp}, the p-value is the likelihood of a permutation value at least as extreme as the mutation value.

		n	N_{mut}	S_{mut}	N/S_{mut}	N/S_{exp}	p-value
nonsense	leaf-specific	2888	247	83	0.205	0.137	0.017
	mosaic	935	7	32	0.219	0.134	0.021
	$clonal_{HISTORIC}$	50	0	2	0.000	0.134	--
	$clonal_{ACCESSION}$	8922	48	306	0.157	0.159	--
missense	leaf-specific	2888	17	83	2.976	2.750	--
	mosaic	935	85	32	2.656	2.758	--
	$clonal_{HISTORIC}$	50	6	2	3.000	2.480	--
	$clonal_{ACCESSION}$	8922	745	306	2.435	2.245	0.007

total	leaf-specific	2888	264	83	3.181	2.877	--
	mosaic	935	92	32	2.875	2.887	--
	$clonal_{HISTORIC}$	50	6	2	3.000	2.606	--
	$clonal_{ACCESSION}$	8922	793	306	2.592	2.404	0.020
